# The role of pharmacy in the management of cardiometabolic risk, metabolic syndrome and related diseases in severe mental illness: a mixed-methods systematic literature review

**DOI:** 10.1186/s13643-021-01586-9

**Published:** 2021-03-31

**Authors:** Dolly Sud, Eileen Laughton, Robyn McAskill, Eleanor Bradley, Ian Maidment

**Affiliations:** 1 School of Life and Health Sciences, Aston University, Birmingham, B4 7ET UK; 2 Pharmacy Department, Leicestershire Partnership NHS Trust, Bradgate Mental Health Site, Glenfield Hospital, Groby Road, Leicester, Leicestershire LE3 9EJ UK; 3 College of Health, Life and Environmental Sciences, University of Worcester, Henwick Grove, Worcester, WR2 6AJ UK; 4 School of Life and Health Sciences, Aston University, Birmingham, B4 7ET UK

**Keywords:** Cardiometabolic, Metabolic, Pharmacy, Pharmacist, Severe mental illness, Monitoring, Screening, Implementation, Guidelines, Cardiovascular disease

## Abstract

**Background:**

Individuals with severe mental illness, e.g. schizophrenia have up to a 20% shortened life expectancy compared to the general population. Cardiovascular disease, due to cardiometabolic risk and metabolic syndrome, accounts for most of this excess mortality. A scoping search revealed that there has not been a review of published studies on the role of pharmacy in relation to cardiometabolic risk, metabolic syndrome and related diseases (e.g. type 2 diabetes) in individuals with severe mental illness.

**Methods:**

A mixed-methods systematic review was performed. Eleven databases were searched using a comprehensive search strategy to identify English-language studies where pharmacy was involved in an intervention for cardiometabolic risk, metabolic syndrome or related diseases in severe mental illness in any study setting from any country of origin. First, a mapping review was conducted. Then, implementation strategies used to implement the study intervention were classified using the Cochrane Effective Practice and Organisation of Care Taxonomy. Impact of the study intervention on the process (e.g. rate of diagnosis of metabolic syndrome) and clinical (e.g. diabetic control) outcomes were analysed where possible (statistical tests of significance obtained for quantitative outcome parameters reported). Quality assessment was undertaken using a modified Mixed Methods Appraisal Tool.

**Results:**

A total of 33 studies were identified. Studies were heterogeneous for all characteristics. A total of 20 studies reported quantitative outcome data that allowed for detailed analysis of the impact of the study intervention. The relationship between the total number of implementation strategies used and impact on outcomes measured is unclear. Inclusion of face-to-face interaction in implementation of interventions appears to be important in having a statistically significantly positive impact on measured outcomes even when used on its own. Few studies included pharmacy staff in community or general practitioner practices (*n* = 2), clinical outcomes, follow up of individuals after implementation of interventions (*n* = 3). No studies included synthesis of qualitative data.

**Conclusions:**

Our findings indicate that implementation strategies involving face-to-face interaction of pharmacists with other members of the multidisciplinary team can improve process outcomes when used as the sole strategy. Further work is needed on clinical outcomes (e.g. cardiovascular risk reduction), role of community pharmacy and qualitative studies.

**Systematic review registration:**

PROSPERO CRD42018086411

**Supplementary Information:**

The online version contains supplementary material available at 10.1186/s13643-021-01586-9.

## Background

Individuals with severe mental illness (SMI) (defined here as bipolar affective disorder, schizophrenia, schizoaffective disorder and other non-organic psychotic disorders) have up to a 20% shortened life expectancy compared to the general population [1]. The majority of deaths in individuals with SMI are due to preventable physical diseases, in particular, cardiovascular disease (CVD) [1], they have a 2-3 times higher risk of dying from CVD when compared to the general population [1]. Evidence suggests that up to 75% of individuals with schizophrenia (versus 33% of the general population) die of CVD [1]. The remainder of deaths is due to unnatural causes, including suicide, homicide and accidents [1]. These data have been well documented in meta-analyses and systematic reviews [2–7]. The mortality gap exists in countries considered to have high standards of healthcare [8] and can in part be accounted for by a higher relative risk (around one- to fivefold) [9] for modifiable cardiometabolic risk (CMR) factors.

CMR is a broad term that describes risk of CVD and diabetes and includes the following: smoking [10–14], overweight/obesity [14, 15], hyperglycaemia [13, 15], hypertension [13, 14, 16], dyslipidaemia [14, 16, 17], and metabolic syndrome (MetS) [18–24]. Public health data from the United Kingdom (UK) [25] and the United States of America (USA) [26] suggest that around two-thirds of individuals with severe mental illness are current smokers, a figure which reflects approximately double that of the general population [25, 26]. A recent systematic review and meta-analysis of 58 studies reported that diet may often be poor [27] in individuals with SMI, literature reviews indicate that overweight and obesity is two- to threefold higher than that in the general population [28, 29].

MetS is a more specific term that describes the concurrence of the most dangerous CVD risk factors [30–33]. MetS is defined by the International Diabetes Federation as central obesity plus any two of the following four factors: raised triglycerides (or specific treatment for this), reduced HDL cholesterol (or specific treatment for this lipid abnormality), raised blood pressure (or treatment of previously diagnosed hypertension) or raised fasting plasma glucose [32]. MetS is one of the most prevalent risk factors for developing CVD in those with SMI [34, 35]. Thirty-seven percent of those with chronic schizophrenia have MetS [18] compared with 24% in the general population [18].

Antipsychotics, used to control psychotic symptoms in people with SMI, are associated with physical side effects including, dyslipidaemia, impaired glucose tolerance and weight gain (more common with newer antipsychotics) [36, 37]; the greatest weight gain has been reported to occur in the first few months of use [36, 38, 39]. Weight gain has also been shown to occur with antidepressants (used to treat negative symptoms in SMI), and mood stabilisers, including valproate/valproic acid and lithium salts [40].

The likelihood of CMR, MetS or related diseases (e.g. type 2 diabetes) is lower in young, drug-naïve individuals and higher in individuals who have severe enduring illness treated with medication (mainly antipsychotics) on a long-term basis. Studies indicate that the CVD associated with MetS in SMI may, to a certain extent, determined by genetic risk factors [41]. In addition to antipsychotic medication, other factors including poor diet, physical inactivity, high rates of smoking, obesity, overweight [42] and inequity to access to and quality of care [43–45] have been reported to contribute. What is not known is the relative contribution of each of these factors.

The availability of rigorous economic data on this subject is limited. In a recent retrospective database review of 57,506 patients with schizophrenia and bipolar disorder in the UK, each incremental CMR factor was associated with an 8.3% and 13.4% increase in total hospital spend respectively [46]. An estimated cost saving of £81 million/year could be made from an investment of £83 million in the physical health of those with SMI in primary care within the UK [47]; this cost saving could rise to £108 million with sustained investment [47].

As far back as 1995, incorporating care for physical health into the care of those with SMI was included in government policies in parts of Australia [48]. In the UK, guidelines for schizophrenia published by the National Institute for Clinical Excellence (NICE) in 2002 [49] outlined recommendations for regular physical health screening. Then, in 2004, as a result of a United States of America (USA) Food and Drug Administration (FDA) warning about the association of antipsychotics and elevated risk of type 2 diabetes, the American Diabetes Association and the American Psychiatry Association published joint guidelines that clearly outlined the need for routine screening for people taking antipsychotics [50]. Within the UK over the past 5 years, much attention has been paid to achieving parity between physical and mental health [51, 52]. The terms screening and monitoring are used interchangably here. 

Despite the convincing evidence for increased CMR, MetS and related diseases in individuals with SMI taking antipsychotic medication as well as explicit recommendations provided by guidelines, screening is often incomplete or inconsistent [53]. A recent review of 39 internationally published studies suggested that rates of routine baseline screening were low (50% only for blood pressure and 59.9% for triglycerides) but less than 50% for cholesterol (41.5%), glucose (44.3%), weight (47.9%) and glycosylated haemoglobin (HbA1c) (< 25%) [53]. Timely and sustained interventions (e.g. lifestyle changes) have been shown to reduce the incidence of CMR, MetS and related diseases and in turn reduce premature morbidity, mortality and disability. Opportunistic and other forms of screening by healthcare professionals are therefore a potentially [54] useful means of detecting risk factors, such as raised blood pressure, abnormal blood lipids and blood glucose.

Systematic reviews have shown that patient interventions delivered by pharmacists, have yielded positive effects on therapeutic, safety and clinical outcomes across different diseases including diabetes and dyslipidaemia [55–58]. These interventions included signposting and advice in relation to health promotion as well as specific risk reduction activities (e.g. smoking cessation) [55–57, 59, 60]. Outcomes include significantly improved mental well-being, reduced risk of disease and premature mortality [55–57, 59, 60].

Literature reviews conducted for those with mental health conditions have shown that pharmacists provide a variety of services and play a significant role in inpatient mental healthcare [61]. Another review including studies from both inpatient and outpatient mental health settings concluded that pharmacists can have a positive impact on outcomes, prescribing practices, patient satisfaction and resource use [62]. Both reviews included those with any type of mental health condition but did not specify a breakdown of specific diagnoses.

We are not aware of any systematic reviews which have specifically explored the nature or impact of pharmacy involvement in CMR, Mets and related diseases (e.g. diabetes, dyslipidaemia) in those with SMI.

Pharmacy staff (e.g. pharmacists, pharmacy technicians) provide services and work collaboratively with patients, informal carers and care professionals to optimise management of illness and disease. This is achieved predominantly through the provision of public health services, e.g. smoking cessation, medicines optimisation, access to medicines such as dispensing and enhanced roles such as independent prescribing [63]. There has not yet been a review of published studies to explore the role of pharmacy/pharmacy staff in managing CMR or MetS and related diseases in SMI. There is a potential for pharmacy to have an impact on morbidity and mortality associated with CMR, MetS, and related diseases in those with SMI.

In 2018, the Royal Pharmaceutical Society of Great Britain published a UK policy document which recommended that the expertise and clinical knowledge of pharmacists must be fully utilised to support people living with mental health problems to ensure they live longer and healthier lives and reduce the mortality gap [64]. This policy included specific reference to the role of pharmacists and pharmacy in relation to  CMR, MetS and related diseases in those with SMI.

There is growing recognition that both qualitative and quantitative evidence can be combined in a mixed-method analysis and synthesis and this can help in understanding how complexity impacts on interventions in specific contexts. In particular, how complex interventions work and for whom, and how the complex health systems into which they are implemented respond and adapt [65].

### Aims and objectives

The primary aim of this systematic literature review is to undertake a detailed analysis and review of the published studies that exist relating to the role of pharmacy or pharmacy staff in CMR, MetS and related diseases in individuals with SMI. This review seeks to undertake an exploration of the range of roles for pharmacy or pharmacy staff as part of interventions relating to CMR, Mets and related diseases, for example, undertaking screening or managing CMR factor or advising on medication that alters CMR risk. This could include, for example, a new or existing pharmacy service or part of an intentional research study intervention (the phrase study intervention will be used here when referring to any of these). Secondary aims are to (i) undertake a review of implementation strategies used in study interventions and their effectiveness in order to inform practice (ii) identify evidence gaps to provide a focus for future research studies.

The objectives are as follows:

1. Identify published quantitative, qualitative or mixed methods studies relating to the role of pharmacy or pharmacy staff in CMR, MetS or related diseases in individuals with SMI
2. Summarise the data and conclusions from those studies
3. Undertake a collective appraisal of that data which will consist of a mapping review and a detailed analysis and review of the implementation strategies used in study interventions that involved pharmacy or pharmacy staff in CMR, MetS and related diseases in SMI
4. Identify limitations and evidence gaps from the studies identified and make recommendations for areas that require further research

## Methods

The Preferred Reporting Items for Systematic Reviews and Meta-Analyses (PRISMA) guidelines [66] were used to standardise the conduct and reporting of the research and the protocol was registered on PROSPERO: CRD CRD42018086411. The PRISMA checklist is attached as Additional file 1.

### Literature search procedure and databases searched

A systematic search was conducted for primary studies in which the study intervention involved pharmacy or pharmacy staff in CMR, MetS or related diseases in SMI. We included any published literature which described an intervention involving pharmacy or pharmacy staff in CMR, MetS or related diseases; this could include, for example, a new pharmacy service or an existing service or part of an intentional research study intervention. Elaborating on what we mean by the term “role”, this could include, for example, pharmacists or pharmacy staff, undertaking screening (e.g. weight checks), managing CMR factor (e.g. providing support for smoking cessation) or advising on medication (e.g. advising on switching medication with lower risk profile for weight gain) (please see Table 1 PICOS criteria for detailed information on these interventions and Additional file 2).

**Table 1:** Participants Intervention Comparator Outcome Study design (PICOS) eligibility criteria for full-text assessment

Category	Inclusion criteria	Exclusion criteria
Participants	Individuals with severe mental illness (SMI) ≥ 18 years. Severe mental illness—bipolar affective disorder, schizophrenia, schizoaffective disorder, psychosis, any psychotic disorder.	Individuals with SMI under the age of 18 years.
Intervention	Pharmacy staff carrying out any of the following activities to any degree: Screening/monitoring for cardiometabolic risk or metabolic syndrome, syndrome X or cardiometabolic disease or related diseases and any of the associated risk factors including lifestyle advice, diet, smoking, alcohol, exercise, cardiovascular disease, diabetes or prediabetes, HbA1c, glucose,weight, BMI, waist circumference, overweight, obesity, lipids, lipid abnormalities, blood pressure and hypertension. Health promotion or risk reduction intervention for cardiometabolic risk or metabolic syndrome, syndrome X or cardiometabolic disease or related diseases or any of the associated risk factors. Medicines management activities relating to the above.	Any of these activities carried out wholly by staff who are not pharmacy staff. Activities that are carried out by pharmacy staff who are not listed.
Comparators (NB it is not compulsory to have a comparator for the study to be included)	Patients with SMI ≥ 18 years who did not receive any intervention. Patients who do not have SMI who have any intervention.	
Outcome	Primary outcome: Change in rate of screening of cardiometabolic risk or metabolic syndrome, cardiometabolic diseases or syndrome X or any of the associated risk factors. Change in health or lifestyle behaviour (risk reduction or health promotion). Diagnosis of metabolic syndrome or identification of individual at high risk of metabolic syndrome. Diagnosis of diseases related to cardiometabolic risk metabolic syndrome including diabetes, cardiovascular disease, hypertension, obesity, overweight, diabetes/high risk of diabetes/pre-diabetes. Change in patient or physical health parameter e.g. BP outcome for the above. Views, perception, opinions, experiences of service users, carers or any care professionals on the role of pharmacy to deliver ANY of the interventions.	Studies that do not measure the primary outcomes.
Study design	Any study design. Any country. Papers written in English only.	No study design will be excluded. Papers not written in the English language.

Database-specific search strategies were developed with assistance from a medical librarian. Eleven electronic databases were searched from inception to January 2018; Medline, EMBASE, PsycINFO, British Nursing Index, AMED, Health Business Elite, Health Management Information Consortium, The Cochrane Library, Health Technology Assessments, Scopus and Web of Science (Additional file 2 provides detailed information on search strategy including, hand and grey literature searches, and Population, Intervention, Comparison, Outcome (PICOS)).

### Study selection process

The eligibility criteria for full-text review are summarised in Table 1 (more detailed information about inclusion and exclusion criteria for studies can be found in Additional file 2: section 1.1). Studies were included if they met the following inclusion criteria: English-language, primary study, published in full, utilising qualitative, quantitative or mixed methods. Only aspects of the studies involving pharmacy or pharmacy staff were extracted for analysis.

**Fig. 1:**
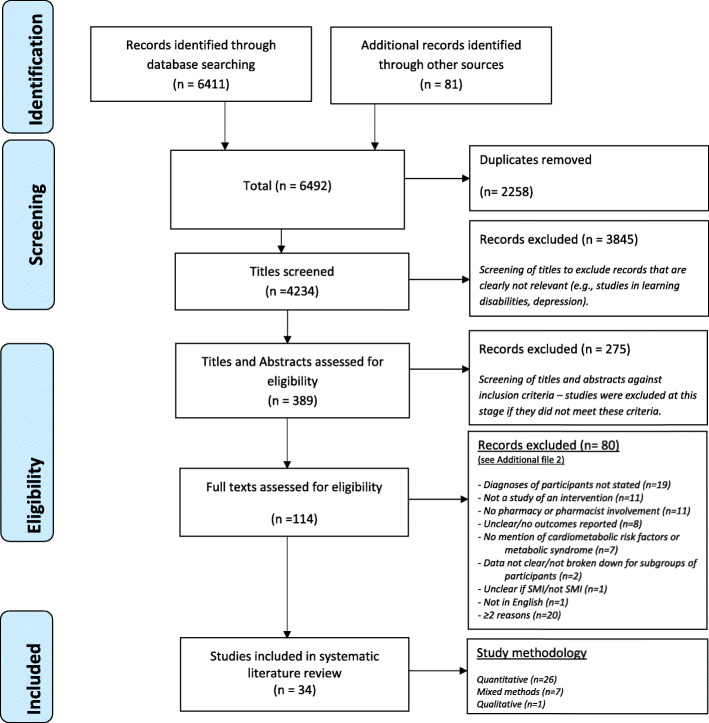
Preferred Reporting Items for Systematic for Systematic Reviews and Meta-Analyses (PRISMA) flow diagram of search results

First, one author (DS) conducted preliminary screening of titles to exclude any publications that were clearly not relevant (e.g. preclinical studies). Second, three authors (DS, EL, RM) independently screened article titles and abstracts against inclusion criteria, to identify potentially relevant studies. Third, three authors (DS, EL, RM) independently reviewed full texts of studies. Consensus on inclusion was reached by discussion between three authors (DS, EL, RM) when necessary, with senior authors (EB or IM) available for arbitration if required (see Additional file 2 Table 1.2 Reasons for excluding studies after full-text review). Please refer to Fig. 1: PRISMA flow diagram of search results for further details.

### Quality assessment

A modified Mixed Methods Appraisal Tool (Additional file 2: 1.3 Quality assessment) was used to assess the quality of included studies [67] by two authors (DS and EL) independently. Consensus on scoring was reached by discussion between authors with senior authors (IM and EB) available for arbitration if required but this was not needed.

Studies were not excluded on the assessed level of quality but the quality assessment process enhanced study interrogation and informed interpretation of the results. In addition one of the main aims of this review was to obtain an overview of all the research conducted in this area, the authors (DS, EL, RM) agreed that exclusion of studies would have potentially resulted in loss of important data.

The quality assessment conducted addressed threats to external validity (e.g. risk of selection bias such as the use of convenience sampling, lack of randomisation, lack of control groups), threats to internal validity (e.g. contamination between the pre and post groups for quasi studies). None of the included studies reported undertaking power analysis calculations to determine the minimum number of participants they required. Please see Additional file 2 for further information.

### Summary of data extraction (study characteristics and results) and collective appraisal

This was carried out in the following steps: summary of study characteristics and conclusions, mapping review and then an analysis and review implementation strategies used in the study intervention. The lead author (DS) utilised a reading support tool (Capti®) to listen to each of the chosen studies three times. Two other authors (EL and RM) read and re-read the included studies. All three authors (DS, EL, RM) independently extracted data regarding information contained within each included study. Discrepancies were resolved through discussion between three authors (DS, EL, RM).

The dataset was heterogeneous for all characteristics including participant characteristics such as definition of SMI and age, study setting, outcomes measured and data collected.

A mapping review [68] (qualitative, quantitative and mixed methods studies) conducted to obtain an overview of the landscape of this particular research area. It also facilitated the identification of trends or themes as well as identification of specific gaps prior to the more detailed analysis and review of implementation strategies used in study interventions [68].

Thirty of the studies included a study intervention that could be classified into one of three categories (i) screening for CMR, MetS or related diseases (ii) screening, identification of risk and implementation of interventions for CMR, MetS and related diseases (iii) implementation of clinical interventions for CMR, MetS and related diseases.

For this review to be meaningful in informing clinical practice, we wanted to gain an understanding of how study interventions were implemented. To understand this ‘how’, we undertook a two-step, detailed analysis and review of the implementation strategies and their effectiveness with regards pharmacy or pharmacy staff in CMR, MetS and related diseases. First, the individual implementation strategies for the study intervention were classified into five categories: ‘Professional’ (e.g. distribution of educational materials, reminders), ‘Organisational’ (e.g. provider-oriented interventions, structural interventions), ‘Financial’ (e.g. provider incentives), ‘Patient-centred’ (e.g. patient education) and ‘Regulatory’ (e.g. peer review). This was done using the Cochrane Effective Practice and Organisation of Care Group (EPOC) taxonomy classification system checklist [69] (Additional file 3) independently by all three authors (DS, EL, RM) with discrepancies being resolved through discussion between three authors (DS, EL, RM).

Second, an analysis of implementation strategies identified was undertaken using the Cochrane EPOC taxonomy classification system checklist [69]; within each category the individual implementation strategies were identified. For example, within the category of ‘Professional’ the individual implementation strategies used to implement the study intervention could be the distribution of educational material or reminders. This was only carried out for those studies where impact of the study intervention could be assessed from quantitative outcome data provided (statistical tests of significance of data obtained for outcome parameters reported by study authors) (e.g. rate of screening for MetS before and after implementation of study intervention). Qualitative data was not analysed for this part of the review. Outcomes were further distinguished as being either a process outcome (e.g. rate of identification of metabolic syndrome) or a clinical outcome (e.g. smoking cessation or weight loss) [70].

The results of statistical tests of significance of data obtained for outcome parameters reported by study authors were used to classify studies into three categories as follows (see Table 7):

↑ or ↓ (bold) statistically significant change in all outcome parameters

^↑^ or ^↓^ statistically significant increase in at least one but not all outcome parameters

= no statistically significant change

## Results

### Study selection and study characteristics

Thirty-four studies were identified (Fig. 1) but the results of two of these were combined [71, 72] and analysed as one study as the findings were both realised from a single research study. The 33 studies showed heterogeneity for all characteristics and outcomes (Table 2). The majority of these (*n* = 25) were quantitative, 4 were qualitative [73–76] and 4 of a mixed methods [77–80] study design. Twenty of these studies included a study intervention where quantitative outcome data allowed for impact to be assessed—statistical tests of significance of data obtained for outcome parameters reported by study authors (a pre-post study design (*n* = 14)) or they compared groups where study intervention was implemented against group where the study intervention was not implemented (a case-control study design (*n* = 5) [78, 79, 81–83] or randomised controlled study (*n* = 1) [84].

**Table 2:** Summary of study characteristics and results

First Author, Year, Country (reference)	Study purpose/objective/pharmacy staff type/setting	Method	Study type	Participant characteristics	Results
MacHaffie^b^, 2002, UK [105]	Sources of health promotion information of those with serious and persistent mental illness obtain Reliability of health promotion information from different sources as perceived by persons with serious and persistent mental illness Pharmacist (not further specified) Mental health outpatient	Quant	Questionnaire and structured interview, descriptive	41 adult (age > 18 years) patients with a 12 month prevalence of serious and persistent mental illness (12 with schizophrenia, 11 schizoaffective disorder, 9 bipolar I, 4 bipolar II, 1 psychotic disorder not otherwise specified (NOS), 3 major depressive disorder, 1 panic disorder) or a lifetime prevalence of these disorders accompanied by evidence that they would have been symptomatic in the last 12 months if it were not for treatment.	Ranked non-psychiatrist physicians, psychiatrists, nurse and pharmacists (in that order) as providing the greatest amount of health promotion information. Reliability of health promotion information in order (highest to lowest) non-psychiatrist physicians, psychiatrists, nurse and pharmacists
Ohlsen, 2005, UK [77]	Nurse-led delivery system: physical problems identified and appropriate treatment and monitoring initiated by prompt referral to suitable specialist services or general practitioners Joint working agreement with other teams including pharmacy put in place prior to starting the service. Pharmacist (not further specified) Community mental health team.	Mixed methods	Pre and post measurements of metabolic parameters and self-esteem. Qualitative description of management issues.	134 adult (18-65 years old) patients with either schizophrenia or schizoaffective disorder.	Results from study relate to the intervention of the nurse advisor. Direct impact of pharmacist was not reported in the findings.
Runcie, 2007, UK [100]	Impact of a protocol for monitoring weight and blood glucose in psychiatric inpatients receiving antipsychotics. Hospital pharmacist. Psychiatric inpatients	Quant	Pre and post measurements: quasi	Adults aged > 18 years .61 patients pre and 59 post intervention with schizophrenia, schizoaffective disorder, persistent delusional disorder, acute and transient psychotic disorder or induced delusional disorder	No significant improvement in recording of admission weight or blood glucose was observed. Ongoing monitoring of weight after admission was significantly more common. For only 29% of patients studied in 2004 was there complete adherence to the protocol.
Barnes, 2008, UK [101]	Quality improvement programme designed to increase screening for the metabolic syndrome in community psychiatric patients prescribed antipsychotics. Hospital chief pharmacist Community mental health team.	Quant	Audit pre and post intervention: quasi	Adults aged > 16 years. Pre intervention: 1616 (82.2%) had psychotic spectrum disorder (International Classification of diseases (ICD)-10 F20-29), 260 (13.2) had bipolar disorder (ICD-10 F30-39), 90 (4.6%) other (not stated). Post intervention: 1277 (84.3%) psychotic spectrum disorder, 182(12.0) bipolar disorder, 54 (3.7%) other.	Measurement or test result was recorded in the clinical records in the previous year (2005): Baseline -BP in 26% of this sample, for BMI (or other obesity measure) in 17%, for plasma glucose (or glycosylated haemoglobin (HbA1c)) in 28% and for plasma lipids in 22%. 1 year after intervention—BP in 43% of this sample, for BMI (or other obesity measure) in 34%, for plasma glucose (or HbA1c) in 38% and for plasma lipids in 35% Predictors of what clinical factors might be related to full metabolic screening: - At baseline, age and a known diagnosis of dyslipidaemia - At 1 year re-audit: known diagnosis of diabetes and type of antipsychotic, relating specifically to clozapine treatment
Taveira, 2008, USA [81]	Compare the efficacy of a pharmacist led cardiovascular risk reduction clinic (CRRC) in the lowering of cardiovascular risk between those with and without mental health conditions. Clinical pharmacist. Primary care clinic.	Quant	Retrospective cross sectional cohort analysis.	Adults aged > 18 years; total of 297 of whom 176 had no mental health condition (MHC); 121 had a MHC of which 92 (76.0%) had a non-severe MHC diagnosis and 29 (24.0%) had a severe MHC (schizophrenia, schizoaffective disorder, bipolar disorder, psychosis not otherwise specified or posttraumatic stress disorder with psychosis).	The mean United Kingdom Prospective Diabetes Study cardiovascular risk score change from baseline is comparable for those without a mental health condition vs those with a non-severe mental health condition and those with a severe mental health condition
Schneiderhan, 2009, USA [89]	Usefulness of a metabolic risk screening program, including point-of-care glucose testing, to quantify baseline metabolic risk in outpatients receiving antipsychotics. Board certified psychiatric pharmacist. Psychiatric outpatient clinic outpatient	Quant	Retrospective, cross-sectional, cohort study	Adults aged > 18 years. Total participants (92) all of whom were on an antipsychotic. Diagnoses were recorded in 88, 53 (60%) schizophrenia or schizoaffective disorder, 18 (20%) bipolar disorder and 17 (19%) major depressive disorder.	63 (71%) met criteria for level 1 metabolic risk (abdominal obesity); of these 63 patients, 38 (60%) met criteria for level 2 risk (abdominal obesity plus hypertension). Patients with a random glucose level greater than 140 mg/dl had a higher likelihood for being at level 2 risk than level 1 risk Women had a significantly higher likelihood for level 1 metabolic risk compared with men African-Americans had a significantly higher likelihood of level 1 risk and BMI greater than 30 kg/m^2^ compared with Caucasians. Patients with a BMI greater than 30 kg/m^2^ had a significantly higher likelihood of diabetes, hypertension, and hyperlipidaemia. Overall, 5 (5%) of the 92 patients met criteria for prediabetes risk
Gable, 2010, USA [90]	Demonstrate the role of pharmacist reviewing the recommendations and interventions a clinical pharmacist made over a 6 month period in an Assertive Community Treatment team by Board certified psychiatric pharmacist Community mental health team	Quant	Retrospective chart review.	Total participants (34)—who had at least one active Axis I Diagnostic and Statistical Manual (DSM)-IV-TR SMI such as schizophrenia, bipolar disorder, or major depressive disorder.	Physical health assessments, review of blood glucose logs, BP undertaken when appropriate (e.g. recent development of diabetes or hypertension). Labs recommended by pharmacist to monitor for adverse effects and disease states (15 times). Coordinate care with other healthcare providers, including those not part of the mental health care team - included recommendations made to primary healthcare providers on non-psychiatric issues including blood pressure, diabetes control (12 times). The interventions/recommendations were part of a study involving a comprehensive medicines management service provided by a pharmacist
Lizer, 2011, USA [102]	Pharmacist assisted psychiatric clinic to improve adherence to medications and quality of life over 6 months. Pharmacist (not further specified) Psychiatric outpatient clinic	Quant	Prospective single centre pilot study: quasi	27 individuals > 18 years with axis I diagnosis: 11 (41%) bipolar disorder 9 (33%) depression, 7 (26%) other (not stated) receiving at least one scheduled medication for mental illness.	**Quantitative** WHOQOL-BREF (abbreviated generic quality of life scale developed through the World Health Organisation) showed statistically significant changes in both the physical capacity Secondary Study Endpoints Overall, there were no significant changes in the metabolic parameters measured except for total cholesterol and low density lipoprotein **Other results** Other pharmacist recommendations included an increase in exercise, education for a decrease in tobacco use. **Qualitative analysis** of pharmacists’ interventions included recommendations (number of times) to: increase exercise to promote weight loss and reduce stress (12), calcium and vitamin D supplementation (12), smoking cessation education (9). Patient self-reported acceptance of these recommendations was exercise (50%); smoking cessation (22%).
Taylor^a^, 2011, UK [73]	Different models for the delivery of clozapine to people with Treatment Resistant Schizophrenia (TRS) Mental health pharmacists (including senior pharmacists) Community mental health team (clozapine clinic)	Mixed method	Prospective.	TRS (any age); 23 patient participant questionnaire 10 patients’ clinic visit observed; 9 interviewed. 23 healthcare professional survey,	Participants in the clinics with a pharmacist reported no difference in health, wellbeing, self-efficacy and ability to manage their own health than clinics without pharmacist input. In terms of the most favourable behaviours: - Doctors scored favourably in 12 of the 19 areas, with nurses and pharmacists equal in 4 of 19 and phlebotomists 2 of the 19. - Pharmacists demonstrated the least favourable consultation behaviours in 8 of the 19 areas, with nurses in 5 and doctors and phlebotomists in 4 of the 19. However when the scores across all 19 domains were averaged the findings demonstrated with greater clarity the participants’ perception of the HCP communication skills within a consultation
DelMonte, 2012, USA [88]	Computerised physician order entry (CPOE) pop-up alert for laboratory metabolic monitoring of patients treated with second generation antipsychotics Clinical psychiatric pharmacist. Inpatient psychiatric unit	Quant	Single-centre, retrospective chart review: quasi	Before and after alert (respectively):62 (36.3%) and 44 (28%) schizophrenia, 43 (25.1%)and 47 (29.9%)depressive disorders, 35 (20.5%) and 39 (24.8%) bipolar disorder, 9 (5.3%) and 11 (7.0%) mood disorder NOS, 6 (3.5%) and 4 (2.5%) personality disorders, 2 (1.2%) and 2 (1.3%) dementia, 2 (1.3%) and 6 (2.8%) anxiety disorders, 5 (2.9%) and 0 substance related disorders, 4 (2.3%) and 1 (0.6%) adjustment disorder, 3 (1.8%) and 2 (1.3%) other. Age > 18 years.	Patients with glucose level available pre-alert 158 (92.4%) and post alert 157 (100) *p* = 0.001. Blood glucose level ordered at the same time as the SGA ordered on the computer system 9 (5.7%) and 31 (19.7%) *p* < 0.0001. Patients with fasting glucose level available (overall) 80 (46.8%) and 110 (70.0%) *p* < 0.0001. Patients with lipid panel available 49 (28.7%) 117 (74.5%) *p* < 0.001. Patients with both glucose level and lipid panel available 47 (27.5%) 117 (74.5%) *p* < 0.0001. Patients with fasting a lipid panel available (overall) 32 (18.7%) 94 (59.9%) *p* < 0.0001 Blood glucose level ordered at the same time as the SGA ordered on the computer system 4 (8.2%) 38 (32.5%) *p* = 0.002.
Koffarnus, 2012, USA [97]	Adherence to American Diabetes Association recommendations for diabetes monitoring following an educational Intervention for physicians in an inpatient psychiatric hospital. PharmD pharmacist. Inpatient psychiatric unit	Quant	Retrospective chart review: quasi	60 patients pre-intervention and 60 patients post intervention with a diagnosis of schizophrenia (3.3% pre and 13.3% post), schizoaffective disorder (33.3% pre and 30% post), bipolar disorder (40% pre and 23.3% post) and major depressive disorder (16.7% pre and 21.7% post)	The physician education program was successful in significantly increasing the assessment of HbA1c values and lipid profiles for patients with diabetes mellitus in a psychiatric institution.
McCleeary-Monthei, 2012, USA [103]	Metabolic monitoring form for antipsychotics initiated by pharmacists and adherence to American Diabetic Association/ American Psychological Association guidelines. Pharmacist (not further specified) Inpatient psychiatric unit	Quant	Retrospective quasi	Pre-intervention total of 33 patients of whom 7 (22%) schizophrenia, 9 (27%) bipolar, 13 (39%) psychoses. Post-intervention total of 30 patients of whom had 6 (20%) schizophrenia, 10 (33%) bipolar, 5 (17%) psychoses. Aged 18-65 years on inpatient ward ≥ 48 h.	In the pre-intervention group. Patients with schizophrenia were significantly more likely to have baseline lipid monitoring. In the post-intervention group in combined data, patients with a diagnosis of diabetes were more likely to have baseline lipid and glucose/HbA1c. All other results were not statistically significant.
Ramanuj, 2012, UK [104]	Implementation of a high-visibility prompt and an educational programme Head of pharmacy. Inpatient psychiatric unit	Quant	Quasi	Total of 36 patients in the first audit cycle of whom 14 (38.9%) schizophrenia/schizoaffective disorder, 5 (13.9%) bipolar disorder, 7 (19.4%) unipolar depression and 7 (19.4%) dementias. Second cycle (after the intervention) total of 38 of whom 12 (31.6%) schizophrenia/schizoaffective disorder, 9 (23.7%) bipolar, 6 (15.8%) unipolar depression, 2 (5.3%) dementias	Glucose and cholesterol levels were monitored at baseline in only 44% and 16%, respectively, of patients in the first audit, although both of these showed significant improvement by the second audit. The proportion of patients in whom random plasma glucose and fasting cholesterol levels were measured 3 monthly after starting antipsychotic medication increased from 41.7% and 25%, respectively, in the first audit to 66.7% for both in the second audit. Baseline and annual monitoring rates for metabolic dysfunction and cardiovascular risk were not significantly affected by the risk profile of the antipsychotic prescribed either in 2008 or in 2010, except for the annual cholesterol monitoring rate, which was paradoxically lower for the high-risk antipsychotics than the all-antipsychotic rate in 2010.
Watkins, 2012, USA [78]	Medication monitoring system based on current guidelines for pharmacotherapy Clinical pharmacist Community mental health team (university based service)	Mixed methods	Analysis of information from database for psychotropic monitoring	68 adults (> 18 years) with a primary diagnosis of schizophrenia or other psychotic disorders limited to schizoaffective disorder or bipolar disorder with psychotic features.	Orders for fasting blood glucose were discontinued and changed to ‘attempt fasting status’ and ‘obtain HbA1c’ and scheduled for every 6 months. Annual lipid panels were changed to every 6 months, if applicable
Kjeldsen, 2013, USA [92]	Outreach visit by clinical pharmacists (providing education to mental health staff) Clinical pharmacist Inpatient psychiatric ward	Quant	Retrospective: quasi	A total of 205 adult (≥ 18 years) patients were included – 93 active implementation and 112 passive dissemination. Individuals with SMI (ICD-10 criteria for schizophrenia (F20.0—20.99) or affective (bipolar) disorder (F30.0—31.99).	A significant improvement of the use of the screening sheet from in the passive dissemination group to active intervention group was found. Consequently, the quality of the screening increased significantly resulting
Cohen, 2014, USA [82]	Follow up study of Taveira—maintenance of glycaemic control and blood pressure control in patients with diabetes following successful completion of a cardiovascular risk reduction clinic Clinical pharmacist. Primary care	Quant	Retrospective	Total of 231 adults, 108 of whom had mental health conditions—breakdown not given for diagnoses	There was no significant difference between diabetic patients with and without mental health conditions in maintenance of HbA1c and systolic blood pressure after discharge from the cardiovascular risk reduction clinic.
Lucca, 2014, India [91]	Adverse drug reactions (ADRs) to antipsychotics and its management in psychiatric patients. Clinical pharmacist. Tertiary care (inpatient) psychiatric hospital	Quant	Prospective interventional study: descriptive	517 patients receiving antipsychotics, of which 89 (29.66%) psychosis, 88 (29.33% bipolar affective disorder, 59 (19.6%) depression, 42 (14%) schizophrenia (22 (7.33%) other diagnoses—not stated))	Approximately 90% of the patients with weight gain (*n* = 30) were enrolled into weight management program (nonpharmacological intervention). If it exceeded 7% of the initial weight after 10 weeks, then switching to another antipsychotic was considered.
Schneiderhan, 2014, USA [84]	Pharmacist comprehensive medication management services using point-of-care tests to reduce of metabolic syndrome risk parameters at 6 and 12 months. Pharmacists qualified who were certified Minnesota medication therapy management services Community – mental health team	Quant	Prospective, multisite, randomised, controlled study.	Total 120 patients (60 received pharmacist intervention and 60 no pharmacist intervention). Anxiety disorders (76.7%, *n* = 89) (including posttraumatic stress disorder [*n* = 12] and obsessive-compulsive disorders [n = 3]), depressive disorders (65.8%, *n* = 79), bipolar disorders (47.5%, *n* = 57), schizophrenia (30.8%, *n* = 37), and schizoaffective disorder (22.5%, *n* = 27).	No statistical differences in metabolic syndrome based on point-of-care tests were observed between the 2 groups (PCS and NCS) at baseline or at 12 months
Barnes, 2015, UK [98]	Programme of screening for the metabolic syndrome in people prescribed continuing antipsychotic medication (Follow on study from Barnes 2008). Hospital chief pharmacist. Community mental health team	Quant	National quality improvement audit: quasi	Adults > 16 years. Total of 1519 patients.72% schizophrenia, schizotypal and delusional disorders, 13% mood/affective disorders, 6% disorders of adult personality and behaviour, 9% unknown or other diagnoses including mental retardation and organic disorders	Over the 6 years of the programme, there was a statistically significant increase in the proportion of patients for whom measures for all 4 aspects of the metabolic syndrome had been documented in the clinical records in the previous year, from just over 1 in 10 patients in 2006 to just over 1 in 3 by 2012. The proportion of patients with no evidence of any screening fell from almost ½ to 1 in 7 patients over the same period.
Bozymski, 2015, USA [83]	Collaborative drug therapy management protocol at a community mental health centre. Board certified psychiatric pharmacist Psychiatric outpatient clinic	Quant	Retrospective chart review: quantitative non-randomised with a control group.	Schizophrenia 49% (*n* = 89) and schizoaffective disorder 23% (*n* = 42). Age > 18 years on an antipsychotic.88% (*n* = 180) from community support services and 12% (*n* = 24) from primary care clinics.	Monitoring of weight, blood pressure, fasting blood glucose and fasting lipid panels was significantly better at the two primary care clinics than the outpatient psychiatric clinic. Family history monitoring took place at 57% of primary care clinic visits was not a statistically significant different. With the limited amount of continuous data obtained, the only statistically significant differences were weight and blood pressure. Waist circumference was not measured or documented at any study visit.
Fischler, 2016, Canada [99]	Clinical practice guidelines National Institute of Health and Care Excellence guideline for schizophrenia. Manager of pharmacy. Inpatient psychiatric	Quant	Retrospective: quasi	Adults with primary diagnosis of schizophrenia or schizoaffective disorder. Number of patients not stated	Adherence to guidance for metabolic monitoring (March 2014, 76.7%; March 2015, 81.6%), Cognitive behaviour therapy for psychosis referral (March 2014, 6.5 %; March 2015, 11. 4 %) and vocational rehabilitation referral (March 2014, 36.6 %; March 2015, 49.1 %) were increased after clinical practice guideline implementation. There was an initial increase in adherence to antipsychotic monotherapy (March 2014, 53.4%; November 2014, 62.7%), which decreased back towards baseline (March 2015, 55.1%).
Lee, 2016, USA [79]	Computerised physician order entry pop-up alert for laboratory metabolic monitoring of patients treated with second generation antipsychotics. Interventions carried out by the psychiatry team to manage metabolic abnormalities found on screening were also identified. (Follow on study from DelMonte 2012). Board certified psychiatric pharmacist. Inpatient psychiatric unit.	Mixed methods	Retrospective chart review	This is a follow on study from DelMonte and reports a third set of results. In this group there were a total of 129 patients of whom 47 (36.4%) schizophrenia, 34 (26.4%) depressive disorders, 21 (16.3%) bipolar disorder, 10 (7.8%) mood disorder NOS, 4 (3.1%) personality disorders, 1 (0.8%)dementia, 6 (4.7%) anxiety disorders, 4 (3.1%) substance related disorders, none with adjustment disorder, 2 (1.6%) other. Age > 18 years.	**Quantitative** No significant decrease in monitoring of glucose levels and lipid panels (fasting or random). Nine patients with abnormally elevated laboratories were identified. Interventions by the psychiatry team included referrals to appropriate healthcare professionals and initiation of medication. **Qualitative** The interventions made by the psychiatry team to manage metabolic abnormalities were not analysed using statistical tests, but instead reviewed and described through a case series format.
McMorris, 2016, USA [74]	Dietary teaching tools for a select population diagnosed with a severe mental illness and limited financial ability. A clinical pharmacist (certified in diabetes management) and a first-year pharmacy resident Community mental health team—assertive community treatment team.	Qual	Questioning and identification of themes. Focus groups	1st phase: 5 Healthcare professionals (mix of psychiatrist, psychiatry resident, clinical social worker, professional counsellor, behavioural health case manager, recovery support specialist, nurse, administrative assistant, clinical pharmacist). Second patients who have a primary diagnosis of schizophrenia, schizoaffective disorder, or bipolar disorder (number of patients not stated).	Phase one: Ten cards were created and distributed to the healthcare professionals (HCPs). A focus group was conducted. HCPs reported the cards were useful in opening dietary choices dialogues and were able to give more specific information on alternative choices. Phase two: From focus group feedback, specific cards for disease states, calorie guidelines, and budget limitations were developed. HCPs immediately utilised them
Porras-Segovia, 2016, UK [93]	Case of an individual with refractory schizophrenia who developed rapid-onset insulin dependence at the commencement of his clozapine therapy. Clinical pharmacist. Inpatient psychiatric unit	Quant	Case report	One individual with TRS	Case report of an individual with refractory schizophrenia who developed rapid-onset insulin dependence at the commencement of his clozapine therapy and in whom diabetes was treated successfully without discontinuing clozapine
Quirk^b^, 2016, UK [75]	Pilot study of process and impact of implementation of the Lester tool (cardiometabolic health resource) in 4 mental health trusts Assess the extent to which the Lester tool may be transferable to other groups of patients. Pharmacy team and pharmacist (not further specified). Psychiatric inpatients/(mental health trust)	Mixed methods	Questionnaire based survey. Focus group	Adult patients with schizophrenia, bipolar disorder or other psychotic disorders. Focus group with 5 service users Questionnaire based survey:195 individuals Focus group: 5 individuals Implementation of electronic tool developed by pharmacy team: 52 patients baseline 29 at follow-up	Qualitative data asked service users various questions about their physical/cardiometabolic health Questionnaire-based survey of inpatients (1) Which health care professional(s) would you speak to if you thought your medication for your mental health was having a bad effect on your physical health? Of 533 only 3 (0.6%) stated pharmacist (2) Where do you get information about how to be physically fit and healthy? Of 564 none stated a pharmacist One hospital NHS trust were involved in a pilot study to implement the cardiometabolic screening tool: (1) Pharmacy department within that hospital developed an electronic tool for collection of cardiometabolic health data. Data entry was completed by ward clerk Informants attributed the shift in the types of interventions offered (e.g. reduction in medication reviews, and increase in offers of advice regarding exercise and diet) to improved confidence amongst ward staff, meaning that they were more likely to offer to intervene themselves, rather than to refer service users to other professionals (doctors or pharmacists). One trust had a Physical Health Strategy Group is the governance group for physical health care Focus group activity with 5 service users (1) To what extent did you feel you were given information about potential adverse physical effects of medication and were empowered to make a decision weighing up the risks and benefits? The mental health trust has a good pharmacy website—but it is not clear how many people are aware of this, and access this.
Shanker, 2016, UK [76]	New model of care—integrated care programme approach review involving both primary and secondary care team members. Clinical pharmacist. Primary care—GP surgery	Mixed methods	Prospective	Individuals on care program approach who have severe mental illness (schizoaffective disorder, schizophrenia, bipolar disorder, drug-induced psychosis). Numbers not given.	No specific outcomes regarding monitoring. Patient feedback about the whole service was positive (waiting time, involvement in decision making, management plan explained)
Bozymski, 2017, USA [87]	Completion of cardiometabolic interventions at a coordinated specialty care clinic through a retrospective chart review of enrolled clients. Psychiatric pharmacist. Community clinic	Quant	Retrospective: descriptive	163 in total - 90 subjects schizophrenia (55.2%), 45 with psychosis not otherwise specified (27.6%), 19 with schizophreniform disorder, (11.7%), and 9 with schizoaffective disorder (5.5%).	One-third of subjects reported tobacco use, and 47 subjects admitted to illicit drug use (primarily marijuana). Nearly one-fourth of subjects also met diagnostic criteria for dyslipidaemia or obesity at some point in the study, with lesser degrees of hypertension and diabetes mellitus; no subjects met criteria for a cardiometabolic abnormality according to baseline data. As a result of the use of the tool the following interventions were made—referral to dietician or health program n = 29 (17.8%), start diabetes medication n = 13 (8.0%), adjust diabetes medication 2 (1.2%), start dyslipidaemia 1 (0.6%). Dyslipidaemia and obesity were (later) found after use of clinical decision support tool found in 37 (22.7%) and 35 (21.5%) clients, respectively
Sud, 2017, UK [86]	Pharmacist led metabolic monitoring service for individuals with severe mental illness. Senior Specialist Mental Health Clinical Pharmacist Psychiatric inpatients. Community mental health team. Early intervention	Quant	Retrospective: quasi	Individuals with severe mental illness schizophrenia, bipolar disorder, schizoaffective disorder, drug induced psychosis and any other diagnosis. Data for inpatient audits 252 patients per year for 3 years 2014-2017, early intervention audit 150 patients per year for 2 years 2016-2017, 900 community mental health patient.	Improvement in rate of screening and monitoring Rate of screening alone in 2013 was 24% (average) Rate of screening and related interventions (total) was 87% as measured 2015 inpatient only In 2016 99% for inpatients and 95% for early intervention team In 2017, 100% for inpatient, 97% for early intervention and 87% for community mental health team patients on care programme approach
Sasson, 2017, USA [94]	Psychopharmacology rounds in a nursing home will decrease overall rates antipsychotic use. This study also measured HbA1c done in past year and lipid panel within 2 years as a secondary outcome. Clinical pharmacist Nursing home	Quant	Prospective single centre: quasi	81 patients in total who were residents at the nursing home, of these 14 had a concomitant diagnosis of dementia and at least one of the following diagnoses: schizophrenia, bipolar, or depression; 31 had dementia and 36 had other diagnoses (not stated).	Metabolic laboratory monitoring improved from 58% (33/57) to 83% (45/54) (*p* = 0.003), however, not broken down for each diagnoses.
Sharma—two publications from one research study (paper and poster), 2018, Australia [71, 72]	Practices and attitudes of Australian mental health practitioners towards assisting their clients to stop smoking and their beliefs about potential Tobacco Harm Reduction strategies for people with SMI. Pharmacist (not further specified) Public and private covering urban and non-urban settings.	Quant	Online, cross-sectional, national survey	267 mental health professionals: Medical practitioners 37 (13.85), Nurses 61 (22.84), Allied health practitioners (occupational therapist, psychologists, pharmacists and social workers) 66 (24.7), community mental health practitioners 74 (22.84), others (not defined) 29 (3.4).	77.5% asked their clients about smoking 66.7% provided health education 31.1–39.7% provided direct assistance 88.4% believed that tobacco harm reduction strategies are effective for reducing smoking related risks 77.9% believed abstinence from all nicotine should not be the only goal discussed with smokers with SMI 56.9% were unsure about the safety 39.3% efficacy of e-cigarettes. Practitioners trained in smoking cessation were more likely to help their clients to stop smoking. Community mental health practitioners and practitioners who were current smokers were less likely to adhere to the 5As (5As = ask, assess, advise, assist, arrange) of smoking cessation intervention. The results of this study emphasise the importance and need for providing smoking cessation training to mental health practitioners especially community mental health practitioners.
Pena, 2018, USA [95]	Pharmacist run metabolic syndrome monitoring clinic Clinical pharmacist Mental health outpatient.	Quant	Pre and post study of metabolic parameter measurements: quasi Survey of Mental health professionals	Referral rate to pharmacist clinic was 24 patients prior to intervention, and 33 patients post intervention. However, outcome data only reported for 17 (51.5%) of the 33 referred post intervention. No breakdown given as to how many have SMI – but authors report that at the facility 85.9% of patients with a diagnosis of schizophrenia had an active prescription for an antipsychotic. 9 mental health professionals completed the survey.	There was a 37.5% increase in overall referral rates to the clinic after intervention, but only 51.5% of patients attended appointments as scheduled. Monitoring of vital signs increased, but monitoring of laboratory parameters decreased. 60% (9 of 15) of providers completed a survey, of which one third indicated they still forget to refer patients to the clinic
Health foundation, 2018, UK [80]	A collaborative project between primary care, community pharmacy and secondary care for physical health checks for those with psychotic illness and provide health coaching for these patients. Community pharmacist (and mention of community pharmacy but not clear if members of staff other than the community pharmacist were involved or not) Community pharmacy	Mixed methods	Results of health screening before and after intervention; patient activation measure (PAM) and health coaching compared to treatment as usual (TAU). Information regarding satisfaction with service collected from patients and care programme approach (CPA) coordinator. Community pharmacists feedback on service provision and benefit to patients	180 patients with psychotic illness were referred to undertake the research pathway/protocol. 10 community pharmacies Number of care coordinators/community psychiatric nurses not stated	70% attended the community pharmacy. 71% of those that attended had all four screening parameters measured (BP, BMI, glucose, lipids) compared to 36% before the intervention was implemented. 100% of patients received health coaching for smoking, exercise and diet (22—stop smoking; 56—exercise; 78—weight loss or healthy eating). PAM questionnaire: 1^st^ appointment 120 patients completed with average score of 52.72; 2nd appointment 41 patients completed with an average score 57.26 and at the 3rd appointment 15 patients completed with an average score of 58.46. 100% of CPA coordinators—data on satisfaction was unclear; 100% of patients agreed/strongly agreed with the time taken to get an appointment and support received. Qualitative data from 4 community pharmacists—data presented could not be used.
Raynsford, 2018, UK [85]	Specialist mental health pharmacist and pharmacy technician on individuals with SMI in primary care (GP practices). Specialist mental health pharmacist and mental health pharmacy technician. GP surgery in primary care	Quant	Prospective	Primary care (GP) severe mental illness registers of 5 GP surgeries were reviewed by pharmacy technicians (total 472 patients). 316 (67%) of these patients were prescribed mood stabiliser or antipsychotics. Pharmacists received referral for 197 patients and undertook interventions for physical health issue (blood tests or electrocardiogram) in 22 of these.	Blood tests were overdue in 16 (73%) cases and out of range in 6 (27%). Out of range and overdue bloods were followed up with the appropriate team. Reasons for overdue include: failure to attend despite requests, patient being out of the country for a long period of time or query regarding whether tests were to be done in secondary or primary care.

Quant: Quantitative. Qual: Qualitative.

^a^Taylor [73], Quirk [75], Shanker [76] and Raynsford [85] included some work and results that were completely irrelevant objectives of this literature review: we will only consider those aspects pertinent to our review question

^b^Asked the opinion/views of an individual with SMI

**Please note that** none of the studies included informal carers of those with SMI

The dataset was heterogeneous for many characteristics including participant characteristics such as definition of SMI and age, study setting, outcomes measured and data collected and did not allow for quantitative data to be pooled or examined by meta-analysis. The authors (DS, EL, RM) used the following methods to analyse the data: (i) a mapping review and (ii) implementation strategies used to implement the study intervention were classified using the Cochrane EPOC taxonomy

Table 3 shows that the most common setting for study intervention was a community mental health/psychiatric outpatient clinic (*n* = 15), followed by psychiatric inpatient wards (*n* = 12). Four studies were based in primary care settings (community pharmacy (*n* = 1)) [80], one in a General Practitioner (GP) surgery [85] and two in a primary care clinic [81, 82]. One study was based in early intervention/psychosis services [86] and one based in other (mix of urban, non-urban and metropolitan centres) [71, 72]. Pharmacists were involved to some extent in delivering the interventions across all studies, and those involved were mostly commonly specialist mental health/psychiatric pharmacists (*n* = 9) [73, 79, 83, 85–90] or clinical pharmacists (*n* = 9) [76, 78, 81, 82, 91–95] (Table 4). Differences in terminology across studies due to differences in country of origin did not allow us to differentiate grades or qualification of pharmacists. Pharmacy technicians were involved in one study [85] and community pharmacists were involved in one study [80]. Two studies made a broad reference to pharmacy team involvement but did not specify particular pharmacy roles [75, 80]. There was a lack of diversity in the country of origin of the studies: 53% (*n* = 18) were conducted in the USA and 35% (*n* = 12) were conducted within the UK (Table 1).

**Table 3:** Summary of settings of included studies

Setting	Number of studies
Mental health outpatient/community mental health team	15
Inpatient	12
Primary care (primary care clinic)	2
General Practitioner (GP) surgery	1
Community pharmacy	1
Early intervention/first episode psychosis services	1
Other (mix of urban, non-urban and metropolitan centres)	1^a^
**Total**	33

^a^Two papers from the same study

**Table 4:** Summary of pharmacy staff type of included studies

Pharmacy staff type	Number of studies^a^
Head of pharmacy/pharmacy manager	4
Specialist mental health pharmacist/clinical psychiatric pharmacist/psychiatric pharmacist	9
Clinical pharmacist	9
Pharmacist	6
Clinical pharmacist with extra /additional qualifications	2
Specialist mental health psychiatric pharmacy technician	1
Community pharmacy	1
Community pharmacy team	1
Pharmacy team	1
1st year pharmacy resident (pharmacist)	1
Hospital pharmacist	1

^a^Total not provided as some studies used more than one staff type in the implementation of study intervention

### Summary of quality assessment

The overall quality of the reported studies, assessed using the Mixed Methods Appraisal Tool (MMAT) [67, 96] was generally good, with twelve studies scoring **** (100%), eight studies scoring *** (75%), and thirteen studies scoring ** (50%) or less. (Additional file 2: 1.3 provides detailed information). Table 5 provides a summary.

**Table 5:** Quality assessment (using modified Mixed Methods Appraisal Tool (MMAT)) [67, 96]

**Qualitative studies**									
		Clear question	Data allows question to be addressed		Relevant sources of data	Data processing relevant	Consideration of context	Reflexivity	Overall assessment of quality
Taylor [73]		✓	✓		✓	✓	✓	✓	****
McMorris [74]		✓	✓		✓	X	✓	X	***
Quirk [75]		✓	✓		✓	✓	✓	X	***
Shanker [76]		✓	✓		X	X	✓	X	*
**Quantitative: randomised controlled trial**									
		Clear question	Data allows question to be addressed		Clear description of randomisation	Clear description of allocation	Outcome data: complete > 80%	Low withdrawal (< 20%)	Overall assessment of quality
Schneiderhan [84]		✓	✓		✓	X	X	X	*
**Quantitative: non-randomised (assessed using modified assessment criteria)**									
		Clear question	Data allows question to be addressed		Recruitment minimises bias	Appropriate measurements	Pre and post groups comparable	Outcome data complete (> 80%) or response rate (> 60%)	Overall assessment of quality
Runcie [100]		✓	✓		✓	X	✓	✓	***
Barnes [101]		✓	✓		✓	✓	✓	✓	****
Schneiderhan [89]		✓	✓		✓	✓	✓	✓	****
Lizer [102]		✓	✓		X	✓	X	X	*
DelMonte [88]		✓	✓		✓	X	✓	✓	***
McCleeary-Monthei [103]		✓	✓		✓	✓	X	X	**
Kjeldsen [92]		✓	✓		✓	✓	✓	✓	****
Ramanuj [104]		✓	✓		✓	✓	✓	✓	****
Sud [86]		✓	✓		✓	✓	X	✓	***
Koffarnus [97]		✓	✓		✓	✓	✓	✓	****
Barnes [98]		✓	✓		✓	✓	✓	✓	****
Fischler [99]		✓	✓		✓	✓	✓	✓	****
Sasson [94]		✓	✓		✓	✓	✓	✓	****
Pena [95]		✓	✓		✓	✓	X	X	**
**Quantitative non-randomised**									
		Clear question	Data allows question to be addressed		Recruitment minimises bias	Appropriate measurements and absence of contamination	Groups comparable	Outcome data complete (> 80%) or response rate (> 60%)	Overall assessment of quality
Taveira [81]		✓	✓		✓	✓	✓	f✓	****
Cohen [82]		✓	✓		✓	✓	✓	✓	****
Bozymski [83]		✓	✓		✓	✓	X	X	**
**Quantitative descriptive**									
		Clear question	Data allows question to be addressed		Sampling strategy relevant	Sample representative	Appropriate measurements	Response rate (> 60%)	Overall assessment of quality
MacHaffie [105]		✓	✓		X	X	✓	X	*
Gable [90]		✓	✓		✓	X	✓	X	**
Lucca [91]		✓	✓		✓	✓	X	✓	***
Porras-Segovia [93]		✓	✓		✓	✓	✓	✓	****
Bozymski [87]		✓	✓		X	X	X	X	***
Sharma [71, 72]		✓	✓		X	✓	✓	✓	***
Raynsford [85]		✓	✓		✓	X	✓	X	**
**Quantitative descriptive**									
		Clear question	Data allows question to be addressed		Sampling strategy relevant	Sample representative	Appropriate measurements	Response rate (> 60%)	Overall assessment of quality
MacHaffie [105]		✓	✓		X	X	✓	X	*
Gable [90]		✓	✓		✓	X	✓	X	**
Lucca [91]		✓	✓		✓	✓	X	✓	***
Porras-Segovia [93]		✓	✓		✓	✓	✓	✓	****
Bozymski [87]		✓	✓		X	X	X	X	***
Sharma [71, 72]		✓	✓		X	✓	✓	✓	***
Raynsford [85]		✓	✓		✓	X	✓	X	**
**Mixed methods studies**									
	Clear question	Data allows question to be addressed	Mixed methods design relevant	Integration relevant	Limitations associated with integration	Assessment of qualitative aspect	Assessment of quantitative aspect	Which aspect achieved lowest score	Overall assessment of quality
Ohlsen [77]	✓	✓	X	X	X	*	***	Qualitative	*
Watkins [78]	✓	✓	X	X	X	*	****	Qualitative	*
Lee [79]	✓	✓	✓	?	X	**	***	Qualitative	**
Health found [80]	✓	✓	✓	X	X	**	***	N/A both equal	**
**Mixed methods studies**									
	Clear question	Data allows question to be addressed	Mixed methods design relevant	Integration relevant	Limitations associated with integration	Assessment of qualitative aspect	Assessment of quantitative aspect	Which aspect achieved lowest score	Overall assessment of quality
Ohlsen [77]	✓	✓	X	X	X	*	***	Qualitative	*
Watkins [78]	✓	✓	X	X	X	*	****	Qualitative	*
Lee [79]	✓	✓	✓	?	X	**	***	Qualitative	**
Health found [80]	✓	✓	✓	X	X	**	**	N/A both equal	**

✓Yes or methodologically sound; X, no or not methodologically sound

?Cannot tell whether methodologically sound or not

Quality Appraisal of Included Studies Score:  *(25%) **(50%) *** (75%) **** (100%)

A limitation was identified amongst those studies which utilised qualitative data—the authors of the studies were not clear about how collecting qualitative data was relevant to answer the research question. Another limitation included either not reporting (*n* = 4) [74, 76–78] or providing a justification for, method of data analysis [79]. Lack of reporting of researcher reflexivity within qualitative studies (*n* = 3) [74–76] and qualitative aspects of mixed methods studies s (*n*=4) [77–80] were also identified as limitations amongst all of these studies.

All four mixed methods studies [77–80] exhibited limitations and scored poorly (50% or less) . These studies were not described by their authors as being ‘mixed methods’, but all included the collection and analysis of both qualitative and quantitative data with the purpose of meeting the overall research objective. Of these, one [79] made reference to the use of mixed data being as being relevant to the research questions. Not unsurprisingly, therefore, all four mixed methods studies scored zero for integration of both qualitative and quantitative data, there was no evidence of findings from different methods being integrated through the results, or any discussion of integration within the published papers.

The randomised controlled study [84] identified for this review scored poorly (25%) due to lack of description of participant allocation, < 80% reporting of outcome data and a high rate participant attrition (> 20%). Quasi experimental approaches were utilised in thirteen [86, 88, 92, 94, 95, 97–104] of the quantitative, outcome studies, (*n*=12) [86, 88, 92, 94, 95, 97–101, 103, 104] of these scored more than 50%. Seventy-one percent [71, 85, 87, 90, 91, 93] (*n* = 5) of the quantitative descriptive studies scored 50% or more.

### Collective appraisal of data: Mapping review (Fig. 2) and detailed analysis and review of the implementation strategies used in study interventions (Table 6 and Table 7)

#### Mapping review (Fig. 2)

##### Role of pharmacy in the healthcare pathway (Fig. 2)

**Fig. 2:**
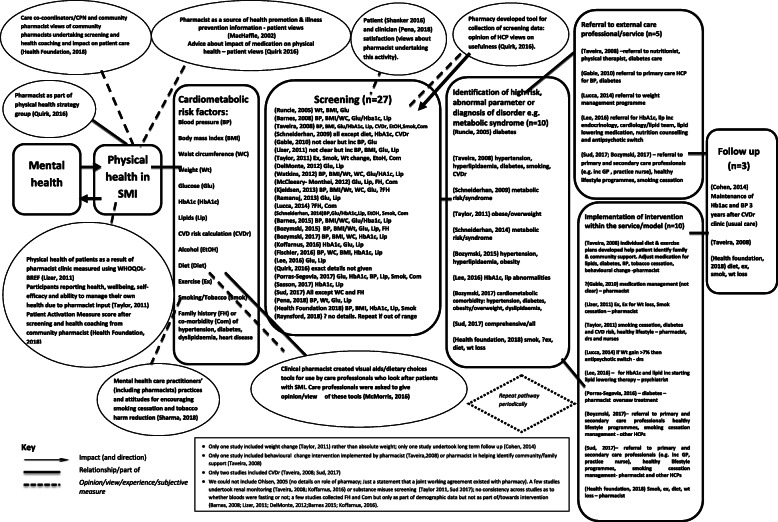
Mapping review

The mapping review included all 33 studies (quantitative, qualitative and mixed methods). Figure 2 shows the key components of the healthcare pathway for CMR, MetS and related diseases and the role of pharmacy at each key component; fourteen studies [73, 79–81, 83, 84, 86, 87, 89–91, 93, 100, 102] included pharmacists in more than one key component. Twenty-seven [75, 79–81, 83, 84, 86, 87, 89–91, 93–95, 97–102, 104] of the 33 studies included direct (e.g. pharmacist undertaking screening such as weight measurements or blood tests) and indirect (e.g. pharmacist writing protocols for other healthcare professionals to use) roles in screening. Ten studies included identification of high risk, abnormal result or diagnosis of disorder, e.g. MetS [73, 79–81, 83, 84, 86, 87, 89, 100]. Ten studies [73, 79–81, 86, 87, 90, 91, 93, 102] included a clinical intervention for health promotion or risk reduction delivered directly by pharmacist to the patient. Five [79, 81, 86, 90, 91] also included pharmacists referring to external care professional/service as a result of identification of risk, abnormal result or diagnosis of disorder.

**Table 6:** Summary of implementation strategies according to Cochrane Effective Practice and Organisation of Care (EPOC) taxonomy

	Screening for CMR, MetS and related diseases				Screening, identification of risk/abnormal result/diagnosis and implementation of clinical interventions for CMR, MetS and related diseases				Implementation of clinical interventions for CMR, MetS and related diseases				Impact assessment possible? ◊
Study, Ref	Patient	Profess	Financial	Organisation	Patient	Profess	Financial	Organisation	Patient	Profess	Financial	Organisation	
**MacHaffie** [105]	**Not an intervention study, questionnaire.**												
**Ohlsen** [77]	**Results of pharmacist input not reported**												**X**
**Runcie** [100]						**✓**							**✓**
**Barnes** [101]	**✓**	**✓**											**✓**
**Taveira** [81]					**✓**	**✓**		**✓**					**✓**
**Schneiderhan** [89]				**✓**									**X**
**Gable** [90]					**✓**	**✓**		**✓**					**X**
**Lizer** [102]					**✓**	**✓**		**✓**					**✓**
**Taylor** [73]					**✓**			**✓**					**X**
**DelMonte** [88]		**✓**											**✓**
**McCleeary-Monthei** [103]		**✓**											**✓**
**Ramanuj** [104]		**✓**											**✓**
**Watkins** [78]	**✓**	**✓**		**✓**									**✓**
**Kjeldsen** [92]		**✓**											**✓**
**Cohen** [82]^a^													**✓**
**Lucca** [91]												**✓**	**X**
**Schneiderhan** [84]					**✓**			**✓**					**✓**
**Bozymski** [83]								**✓**					**✓**
**Barnes** [98]	**✓**	**✓**											**✓**
**Fischler** [99]		**✓**											**✓**
**Lee** [79]						**✓**							**✓**
**McMorris** [74]										**✓**			**X**
**Porras-Segovia** [93]										**✓**			**X**
**Quirk** [75]	**Pharmacy designed tool for recording data by other staff, however, exact details unclear**												
**Shanker** [76]								**✓**					**X**
**Koffarnus** [97]		**✓**		**✓**									**✓**
**Bozymski** [87]						**✓**							**X**
**Sud** [86]						**✓**	**✓**	**✓**					**✓**
**Sasson** [94]				**✓**									**✓**
**Sharma** [71, 72]	**Not an intervention study, survey**												
**Pena** [95]	**✓**	**✓**	**✓**	**✓**									**✓**
**Health foundation** [80]					**✓**	**✓**		**✓**					**✓(for 2 of 4 outcomes measured)**
**Raynsford** [85]				**✓**									**X**

Please note none of the studies included a regulatory strategy.  Prof: Professional

^a^Although this study did not involve implementation of an intervention it was included as it provided follow up data for a study that did

◊If data were provided to assess impact of the intervention before and after or to a comparator group or described impact over a period of time

**Table 7:** Detailed analysis of the 20 studies that included outcome data that allowed for assessment of impact of intervention^a^ (1 = used, 0 = not used)

Domain	First author (reference)		Runcie [100]	Barnes [101]	Taveira [81]	Lizer [102]	DelMonte [88]	McCleeary-Monthei [103]	Ramanuj [104]	Watkins [78]	Kjledsen [92]	Cohen [82]	Schneiderhan [84]s	Bozymski [83]	Barnes [98]	Fischler [99]	Lee [79]	Koffarnus [97]	Sud [86]	Sasson [94]	Pena [95]	Health found [80]	% Using strategy
**Professional**	1	**Educational materials**	1	1	0	0	0	0	1	0	1	0	0	0	1	0	0	1	1	0	1	1	**45**
	2	**Educational meetings**	0	1	0	0	0	0	1	0	0	0	0	0	1	0	0	1	1	0	0	0	**25**
	3	**Local consensus process**	1	0	0	0	0	0	0	0	0	0	0	0	0	1	0	0	0	0	0	0	**10**
	4	**Educational outreach visits**	0	0	0	0	0	0	0	0	1	0	0	0	0	0	0	0	0	0	0	0	**5**
	5	**Local opinion leaders**	0	0	0	0	1	0	0	0	0	0	0	0	0	0	0	0	1	0	0	0	**10**
	6	**Patient mediated interventions**	0	0	1	1	0	0	0	0	1	0	0	0	0	0	0	0	1	0	0	1	**25**
	7	**Audit and feedback**	0	1	0	0	0	0	1	0	0	0	0	0	1	1	0	0	1	0	0	0	**25**
	8	**Reminders**	0	0	0	0	1	1	0	1	0	0	0	0	0	0	1	0	1	0	1	0	**30**
	9	**Questionnaire**^b^	0	1	0	0	0	0	0	0	0	0	0	0	1	0	0	0	0	0	0	0	**10**
**Financial**	10	**Provider incentive**	0	0	0	0	0	0	0	0	0	0	0	0	0	0	0	0	1	0	0	0	**5**
	11	**Patient**	0	0	0	0	0	0	0	0	0	0	0	0	0	0	0	0	0	0	1	0	**5**
**Patient**	12	**Educational materials**	0	1	0	1	0	0	0	0	0	0	0	0	1	0	0	0	0	0	1	0	**20**
	13	**Reminder card**^b^	0	1	0	0	0	0	0	0	0	0	0	0	1	0	0	0	0	0	0	0	**10**
	14	**Questionnaire**^b^	0	1	0	0	0	0	0	0	0	0	0	0	1	0	0	0	0	0	0	0	**10**
	15	**Behaviour change**	0	0	1	0	0	0	0	0	0	0	0	0	0	0	0	0	0	0	0	1	**10**
	16	**Education—face to face**	0	0	1	1	0	0	0	1	0	0	1	0	0	0	0	0	0	0	0	1	**25**
	17	**Medication change** ^b^	0	0	1	0	0	0	0	0	0	0	?1	0	0	0	0	0	0	0	0	0	**5(?10)**
	18	**Facilitate adherence to medicines**^b^	0	0	0	1	0	0	0	0	0	0	0	0	0	0	0	0	0	0	0	0	**5**
	19	**Identification of family support**^b^	0	0	1	0	0	0	0	0	0	0	0	0	0	0	0	0	0	0	0	0	**5**
**Organisational**	20	**Revision of professional role**	0	0	1	0	0	0	0	0	0	0	1	0	0	0	0	0	1	0	0	0	**15**
	21	**Clinical MDT team**	0	0	0	0	0	0	0	0	0	0	0	1	0	0	0	0	0	1	0	1	**15**
	22	**Continuity of care**	0	0	1	1	0	0	0	1	0	0	0	0	0	0	0	0	1	0	0	0	**20**
	23	**Medical systems records change**	0	0	0	0	0	0	0	0	0	0	0	0	0	0	0	1	1	0	0	1	**15**
	24	**Changes in equipment**	0	0	0	0	0	0	0	0	0	0	0	0	0	0	0	0	0	0	1	0	**5**
**Total number of implementation strategies used**			2	7	7	5	2	1	3	3	3	0	2(?3)	1	7	2	1	3	10	1	5	6	
**Process outcomes**	**Screening**		^↑^	↑			**↑**	**=**	^↑^	**?**			=	**↑**	**↑**	^↑^^c^	**=**	^↑^	**↑**	**↑**	^↑/↓^	^↑^^c^	
	**Rate of diagnosis of MetS**										**↑**		=										
	**Mean number of abnormal metabolic parameters as part of MetS**												=										
	**Advice about smoking, diet/healthy eating, exercise,**																					^↑^^c^	
**Clinical outcomes**	**Physical health domain of WHOQOL-BREF**					**↑**																	
	**Improvement in blood results e.g. reduced lipids**					^↑^																	
	**Time to achieve same outcome in CVDr as general population**				↑																		
	**Maintenance of HbA1c and BP after discharge from CVDr clinic**											=											
	**Improved PAM score**																					^↑^^c^	

^a^If data were provided to assess impact of the intervention before and after or to a comparator group or described impact was assessed over a period of time

^b^Not explicitly listed in EPOC classification but agreed between authors (DS, EL, RM)

^c^No statistical tests of significance undertaken

?Unclear

WHOQOL-BREF (abbreviated generic  quality of life questionnaire  developed through the World Health Organisation), the physical health domain includes: activities of daily living, energy and fatigue, mobility, work capacity.

PAM (Patient Activation Measure): a 22-item measure that assesses patient knowledge, skill, and confidence for self-management

**↑** or **↓** (**bold**) statistically significant change in all outcome parameters

^↑^ or ^↓^ statistically significant increase in at least one but not all outcome parameters

= No statistically significant change

##### Role of pharmacy in other activities

The mapping review also shows pharmacists roles in other activities. This includes a pharmacist as part of a physical health strategy group [75] and clinical pharmacists creating visual aids/dietary choices tools for use by care professionals who look after patients with SMI [74].

##### Gaps in the evidence base

As well as identifying the key components of the healthcare pathway where pharmacy have been involved, the mapping review also highlighted important gaps in the published evidence base where little or no literature was found: screening of waist circumference and weight/weight change, cardiovascular and diabetes risk assessment using formal risk assessment tools/calculators, role/involvement of community pharmacy or pharmacy staff (e.g. pharmacy technicians) within primary care, follow-up of individuals after implementation of a study intervention, utilisation of behaviour change techniques or community or family support, and finally the views/perceptions/experiences of patients, (their) informal carer or caregiving dyads and care professionals where qualitative data synthesis had been undertaken. Finally there was no specfic mention of high dose or simultaneous use of two or more (also known as polypharmacy which often results in high doses of antipsychotics) or switching antipsychotics based on risk of CMR, MetS and related diseases. 

##### Assessment of weight gain

Weight gain was part of the screening undertaken by a pharmacist in two studies [73, 91]; in one study around 75.7% of patients who attended pharmacist-led clozapine clinic [73] gained weight since starting clozapine. In the other study [91], weight gain (*n* = 30) was the most commonly observed adverse drug reaction observed by the pharmacist. The latter study [91] also included ≥ 7% weight gain as a trigger for referral to dietary support and antipsychotic switch as recommended in current guidance [36].

##### Application of risk assessment tools/calculators

Three studies [81, 82, 86] included CVD risk assessment and none included diabetes risk assessment using formal tools/calculators. Fifteen percent of the studies [78, 87, 92, 99] included waist circumference as an outcome measure—this may reflect a lack of understanding of its importance as a predictor of CVD or lack of inclusion in guidelines on which study protocols were based. Taveira [81] concluded that patients with diabetes and with mental health conditions (MHCs) achieve the same CVD risk reduction (using a formal CVD risk assessment calculator as an outcome measure) as those without MHCs. The duration of enrolment with risk reduction clinic required to achieve this was around 25% longer than those without MHCs.

##### Follow up of patients after completion of the study intervention

We identified one study [82] that reported findings of follow up of patients after study intervention had been completed [81]; these patients received usual care between the study intervention and the point of collection of outcome data for follow up. This study concluded that there was no significant difference in the duration of maintenance of blood pressure and HbA1c up to 3 years after people with diabetes with MHCs were discharged from a pharmacist-led cardiovascular risk reduction clinic [82]. The authors point out that their model of care was effective for treating particular specific aspects of CMR or MetS or related diseases in patients with and without MHCs but that more work is needed for specific mental health conditions and whether further benefits could be gained by treating both MHCs and physical health conditions concurrently. This study did not provide detailed breakdown of outcomes for those with SMI (instead of reporting results for those with SMI under the general heading of MHCs which included a mix of diagnoses: schizophrenic disorder, episodic mood disorders including depression, bipolar disorder, depressive disorder and anxiety).

### Detailed analysis and review of implementation strategies of study intervention using the EPOC taxonomy (Tables 6 and 7)

#### Studies that included quantitative data that allowed for assessment of impact of study intervention (study authors had undertaken statistical tests of significance of data obtained for outcome parameters)

Table 6 shows a summary of the implementation strategies used in the study intervention using the EPOC taxonomy as well as which of these allowed for assessment of the study intervention to be assessed. Detailed analysis of the 20 studies that included quantitative outcome data (classified as being process or clinical) that allowed for assessment of the impact of the study intervention is shown in Table 7 (authors reported quantitative data for groups being compared, e.g. study intervention vs no study intervention and statistical tests of significance of data obtained for outcome parameters reported by study authors). These studies were published between 2007 and 2018. The most frequently used implementation strategies identified using the EPOC taxonomy [69] were those orientated towards healthcare professionals and patients. Of the healthcare professional-oriented implementation strategies, distribution of educational materials (published or printed recommendations for clinical care, including clinical practice guidelines for CMR or MetS or related diseases) was the most commonly used (45%) (*n* = 9) [80, 86, 92, 95, 97, 98, 100, 101, 104]. Reminders (e.g. computer pop-up alert) was the next most commonly used strategy being applied in 30% (*n* = 6) [78, 79, 86, 88, 95, 103]. With regards patient-orientated interventions, the use of face-to-face education, educational materials, reminder cards and questionnaires was applied in 25% (*n* = 5) [78, 80, 81, 84, 102], 20% (*n* = 4) [95, 98, 101, 102], 10% (*n* = 2) [98, 101] and 10% (*n* = 2) [98, 101] of studies respectively. Two finance-orientated interventions (provider incentive [86] (UK) and patient incentive [95] (USA)) were used in any of the studies.

The total number of implementation strategies used varied from 0 to 10 per study. The overall median number of implementation strategies used per study was three. Sixteen studies reported process outcomes only, three [81, 82, 102] clinical outcomes only, and one [80] both process and clinical outcomes. The relationship between the total number of implementation strategies used and the impact on the outcomes measured is unclear. The quality assessment data for these studies is reported below.

##### Process outcomes

Process outcomes included screening for CMR or MetS or related diseases and rate of identification of MetS. In those studies in which there was a statistically significant improvement in process outcomes (*n* = 7), 50% used 7 or more implementation strategies [86, 98, 101], 60% used educational materials [86, 92, 98, 101], 50% used educational meetings [86, 98, 101], and 50% used audit and feedback [86, 98, 101], all targeted at healthcare professionals. Studies using a smaller number of implementation strategies (3 or less) [88, 92, 94] also reported achieving significant improvement in process outcomes; all of these had one thing in common—some form of face-to-face contact between healthcare professionals (pharmacist-led multidisciplinary team) [94], educational outreach [92] and local opinion leaders [88]. Including pharmacists alone as part of the clinical MDT team as the sole implementation strategy resulted in significant improvement in process outcomes in two studies [83, 94].

Reminders (e.g. pop up alerts on computer systems) were a frequently used implementation strategy within the studies. Despite this, their use alone does not appear to be associated with significant improvement in process outcomes. In one study, a pharmacist produced a template reminding clinicians to undertake screening and attached this to the medication charts of patients with SMI who needed screening [103]; this had no impact on the rate of screening. In a study by DelMonte et al. [88]; a pharmacist-designed computer pop-up alert and ‘champion psychiatrist’ formed part of an intervention to improve the uptake of blood tests for CMR or MetS or related diseases: they found that the majority of blood tests were ordered at the same time as the pop-up alert. However, a follow-up study conducted a few years later using the pop-up alert alone [79] revealed a statistically significant decline in the number of blood tests ordered within 24 h of the reminder; suggesting that the champion psychiatrist was the more effective aspect.

All of the studies in which there was a statistically significant improvement in all process outcomes scored ***(75%) or more in the quality assessment except one [83] which scored **(50%). Where studies were mixed methods that studies the quality assessment score for the quantitative aspect of the study has been quoted here as this part of the review was specifically concerned with quantitative data.

##### Clinical outcomes

Three studies investigated the impact of study interventions on clinical outcomes only [81, 82, 102] two of which [81, 82] scored **** (100%) and the other [102] * (25%) in the quality assessment. Two of these three studies were linked to each other in that one [82] was a follow-up study of the other [81]. We included the follow-up study [82] in our review despite the fact that it did not directly include the implementation of a study intervention; this 3-year observational study provided some valuable data and insight on follow up and long term impact of the pharmacist intervention (cardiovascular risk reduction clinic) included in the first study [81]. The follow-up study [82] found that there was no significant difference between diabetic patients with and without mental health conditions (including those with SMI) in maintenance of diabetic control (as measured by HbA1c) and systolic blood pressure in the 3 years after discharge from the cardiovascular risk reduction clinic.

In the other two studies [81, 102], patient-mediated strategies and face-to-face patient education were implementation strategies that where both utilised. One of these studies reported a significant improvement in a measure of physical well-being (WHOQOL-BREF) [102] and the other reported that a 25% longer (statistically significant) enrolment time in the research study was needed to achieve the same outcome in CVD risk reduction in diabetic individuals with mental health conditions compared with those without [81].

##### Study intervention with a reduction in process outcome

One study reported a reduction in the rate of screening for CMR or MetS or related diseases [95] was conducted over a 4-month period. This study scored ** (50%) as part of the quality assessment.

##### Process and clinical outcomes

We identified one study [80] which looked at both process and subsequent clinical outcomes. This study did not include any statistical analyses or statistical tests of significance of outcome data collected and scored ** (50%) as part of the quality assessment.

## Discussion

The primary aim of this systematic literature review was to undertake a detailed analysis and review of the published studies that exist exploring the role of pharmacy, or pharmacy staff in CMR or MetS or related diseases in individuals with SMI.

The majority (81% (*n*=27) of studies) of published evidence exists, for specialist mental health or clinical pharmacists involvement in screening either directly (e.g. undertaking blood pressure, blood cholesterol measurements) and indirectly (e.g. writing protocols for other healthcare professionals to use) based in community mental health/psychiatric outpatient clinics (45% of studies (*n* = 15)) as well as inpatient settings (36% (*n* = 12)). Some evidence exists, 30% (*n* = 10), for pharmacist’s involvement in identification of individuals at high risk for diagnosis of CMR, MetS or related disease and in the provision of a clinical intervention for health promotion or risk reduction, 30% (*n* = 10), (e.g. pharmacological interventions for management of hypertension, type 2 diabetes, hyperlipidaemia). Almost 42% of studies included a pharmacist in the study intervention at all key components of this healthcare pathway from screening, through to identification of high risk, abnormal parameter or diagnosis of disorder, e.g. metabolic syndrome and then implementation of clinical intervention.

Sixty percent (*n* = 20) of studies included quantitative outcome data (process or clinical) that allowed for assessment of the impact of the study intervention. Of these, 55% (*n* = 11) included a pharmacist undertaking screening and 40% (*n* = 8) [79–81, 84, 86, 87, 100, 102] included a pharmacist at all key components of the healthcare pathway. Of those 20 studies 35% (*n* = 7) [86–88, 92, 94, 98, 101] reported statistically significant improvement in all process outcomes (e.g. rate of diagnosis of MetS) and 10% (*n* = 2) [81, 102] in all clinical outcomes measured (e.g. physical health domain of WHOQOL-BREF).

Factors that facilitate specialist mental health or clinical pharmacist involvement in screening of CMR, MetS and related diseases in those with SMI may include being part of and having a clearly defined role within a multidisciplinary team; access to appropriate resources; effective engagement with those with SMI; effective collaboration with multi-disciplinary team/management within healthcare settings to facilitate set up and roll out of services; clinical knowledge, skills and training (e.g. taking bloods and ordering lab measurements); systematic approach (e.g. application of standardised care) and trusted member of healthcare team and enhanced roles that include prescribing.

Very little evidence currently exists for the role of community pharmacists or other pharmacy staff (e.g. pharmacy technicians) within primary care settings. This data primarily comes from the USA and the UK. This finding has particular relevance within the UK, where up to a third of people with SMI are treated solely in primary care [106]. Even in countries with very well developed secondary psychiatric care systems (including the UK) the role of primary care is key [107]. Accessibility is a known social determinant of health [108]. Recent work has shown that 90% of the population can access a community pharmacy within a 20 min walk from where they live [109]. Individuals who experience the highest rates of deprivation, which includes those with SMI, could benefit the greatest from this level of access [108]. The use of high dose or polypharmacy are associated with higher risk of CMR, MetS and related diseases [1, 40] - no specific mention was made of these in any of the studies. Switching to antipsychotics with a lower risk of CMR, MetS and related diseases represents a potentially useful approach [36, 37], again nothing specific was found in any of the studies. These represent gaps in the evidence base.

Clinical outcomes were reported for two studies where pharmacists were involved in activities other than screening. So, pharmacy could have a role to play beyond screening and towards identification of risk, abnormal result, diagnosis and implementation of clinical interventions for CMR, MetS and related diseases. There is a lack of data and studies on clinical outcomes and studies that examine the link between specific process outcomes (e.g. screening for diabetes using glycosylated haemoglobin/HbA1c) and subsequent improvements in clinical outcomes (e.g. improved diabetes risk or control, diabetes risk calculators, cardiovascular risk calculators) in those with SMI.

Very little evidence, however, was found for their involvement in screening for weight, weight gain or change or waist circumference. Surprising in light of the fact that systematic reviews show that the prevalence of overweight and obesity is two- to threefold higher in those with SMI than that in the general population [28]. A recent study conducted in North America found nearly 80% of a sample of over 10,000 people with diagnoses of SMI to be overweight or obese [110]. Another systematic review found that waist circumference enables prediction of MetS with a sensitivity of 79.4% and a specificity of 78.8% [111]. The IDF emphasises the importance of waist circumference as a mandatory feature of MetS [32]. Weight, weight gain and waist circumference should be included in any screening intervention involving pharmacists given the increased prevalence and usefulness in predicting MetS.

Improving the physical healthcare for those with SMI is a key component of current mental health guidelines, policies and commission documents across the world [112–116]. A recent cross-sectional study of 5091 patients with schizophrenia in secondary care psychiatric services [117] found low rates of clinical interventions for blood pressure (25.2%), cholesterol levels (19.9%) and glucose levels (53.5%) and smoking (57.2%) where screening indicated a need. They are also less likely to receive treatment for cardiovascular conditions [118] or diabetes [119]. This represents a potential opportunity for pharmacy to become involved particularly as this review has shown the significant and positive impact of specialist mental health pharmacist/clinical pharmacists on process outcomes (i.e. screening).

Where qualitative data was collected, this was not synthesised by the study authors using any qualitative synthesis methods and where it was the method of synthesis was not justified. In addition, researcher reflexivity was not reported; there was no examination or critique of how the researcher impacted on the study or the participants for this data. As a result of this and the heterogeneity that existed within the studies that collected qualitative data utilisation of qualitative data in our systematic review was limited (e.g. could not be integrated with quantitative data).

We were able to use this qualitative data as part of our mapping review. Qualitative research has the potential to make significant contributions to health services and policy research. It provides valuable insights into the ways that health is conceptualised, experiences of health and illness, dynamics of multi-disciplinary teams and numerous aspects of care delivery [120]. In addition, the potential value that the mixed methods approach of this review has not been able to be fully realised.

Three quarters of the studies that utilised a mixed-methods approach did not explain how this approach was relevant to the answering research question and all mixed methods studies failed to integrate the quantitative and qualitative data collected. In the absence of integration, the knowledge harvested is only equivalent to the sum of that derived from a qualitative study and a quantitative study undertaken separately, rather than achieving a “whole greater than the sum of the parts” [121]. A lack of robust mixed methods studies exist on this topic.

High-quality RCT data is lacking. Numerous good quality studies quantitative studies exist which utilise non-randomised (mainly quasi) and descriptive approaches. These quasi studies were performed at population level and may therefore have included individuals who may otherwise have been excluded from RCTs, e.g. severely unwell. Quasi studies are also viewed as being more pragmatic evaluating the real-world effectiveness of a study intervention implemented by clinical staff, rather than by research staff under research conditions [122]. Therefore, quasi-experimental studies may also be more generalisable and have better external validity than RCTs [122]. However, bias can occur in these types of study leading to a threat to internal validity (e.g. differences between active and control groups are not accounted for) [122].

Secondary aims were to undertake a detailed analysis and review of implementation strategies used in study interventions and their effectiveness to inform practice and to identify evidence gaps to provide a focus for future research studies. Where impact of the study intervention could be assessed from quantitative outcome data, it was not clear from significant proportion of those studies how the total number or type of implementation strategies were selected or decided upon to facilitate the implementation of the study intervention. None of the studies had any fidelity measures [123] nor was it clear to what extent the strategy was implemented in practice. Three studies discussed the process of identifying barriers in clinical practice [95, 98, 101] with subsequent implementation strategies being developed/chosen to target these barriers in one study [95]. In one study, the authors acknowledge that information on the actual implementation of the change intervention was not collected from the health services that participated [98].

The duration of time over which the study was conducted is also important. The study that showed a reduction in the rate of screening for CMR or MetS or related diseases [95] was conducted over a 4-month period. The most likely explanation for this reduction in the rate of screening is that current guidelines recommend annual screening. This represents a fault in study design for that study rather than a positive or negative outcome of the study; it was not conducted over a clinically appropriate duration of time.

The relationship between the total number of implementation strategies chosen and the subsequent impact on outcomes measured is unclear for either process outcomes such as blood tests or clinical outcomes such as improvement in lipid results. What may be more important is the specific type of implementation strategies chosen (in other words the specificity of implementation strategy chosen is important). With regards process outcomes, the following strategies appear to be particularly effective: educational materials, educational meetings, clinical audit and feedback and any strategy that uses face-to-face interaction between healthcare professionals. The use of multiple strategies (> 1 strategy) carries with it an inherent problem in determination of causality and then the effectiveness of individual implementation strategies when more than one is used. Any overlap, repetition, synergy or hindrance that may occur as a result is also difficult to determine. Similar to other reviews, we were unable to find any study where head-to-head comparison of different implementation strategies was undertaken [70, 124]. We have shown here that the role of face-to-face interaction, such as a pharmacist-led multidisciplinary ward round or pharmacist outreach visits is a specific aspect that is important in achieving a statistically significant impact on both process and clinical outcomes.

### Strengths

This systematic literature review used a robust systematic search strategy and data was appraised using validated tools and methodology. In addition, assessment of methodological quality, mapping review and assessment of the implementation strategies was carried out and checked independently by three authors using an internationally recognised taxonomy [69]. The inclusion of all types of study (qualitative, quantitative and mixed methods) is also a major strength and reflects that studies and data/outcomes of all types contribute to our understanding of this area of clinical practice.

The authors of this review have important experience which is directly relevant to the area of research of this review. DS, EL, RM and IM have extensive experience working as practising clinical pharmacists within multidisciplinary teams. DS, RM and IM also have extensive experience of working within mental health settings and both IM and EB have extensive experience of conducting applied research within mental health settings.

The mapping review was conducted as a mixed-methods review; this facilitated the identification of trends or themes as well as identification of specific gaps which would otherwise not have been possible if we had only used either qualitative or quantitative studies.

### Limitations

There are limitations at two levels within this review; limitations at individual study level included in the in results and discussion and more general limitations with this approach. Outcomes reported by studies may have been impacted up on by factors external to the study protocol such as concurrent healthcare or quality improvement programmes, initiatives or healthcare staff that distracted or raised awareness of the study intervention. This was acknowledged in some of the studies [79, 88, 98]. Improvements in outcomes reported may have been an artefact arising from improved documentation or systems rather than the intervention itself. Conversely, where less effective outcomes were found, this may be due to data collection issues such as inability to access records outside the study setting.

Interpretation of the association between the implementation strategy, improved process and/or clinical outcome is not possible without being able to assess the effort that was put in to putting these into practice as it was not reported—authors of the studies did not provide detailed description of how the strategies were implemented or to what extent.

A limitation of the use of any taxonomy is that the results presented show our interpretation of the main method of delivery as described by the authors of the study under review; some interventions cannot be delivered in a mutually exclusive fashion. For example, classification of a study as patient-orientated intervention using “educational materials” could not have been completely free from “face to face education” by the healthcare professional who gave these materials to the patient.

Other limitations of note include the following:

1. Heterogeneity in healthcare setting, outcome measures chosen and timing of intervention—this makes it difficult to interpret what works for whom in what circumstances
2. Detailed exploration of and consideration was given to possible ways in which the studies could be compared; however, heterogeneity of aims, study design/models delivered, population demographics, data collected and outcomes measured prevented integrated quantitative synthesis/data pooling (e.g. meta-analysis). Our review relied wholly on statistical analyses carried out by study authors
3. Studies where outcomes were recorded under group headings rather than specific groups, for example, pharmacy staff within healthcare professionals group [71, 72], and those with SMI within all individuals with mental health conditions [81, 82]
4. Lack of reporting of patient diagnosis; 19 studies were excluded purely on the basis that diagnoses were not stated—potentially important data or information about the role of pharmacy may have been lost, e.g. pharmacists role in medicines optimisation of antipsychotics
5. The search was restricted to articles published in English, it is therefore possible that we failed to retrieve all studies that may have been eligible for addressing our research question
6. Some of the studies identified for this review reported the results of audits conducted within healthcare settings. Within some of these audits, the audit criteria allow for refusal or decline by the patient (e.g. refusal to have a blood test) to be recorded in outcome data as being compliant (i.e. the same as someone having a blood test). However, where research studies are conducted, a refusal would be regarded as attrition. As such this may have resulted in an overestimation of the effect of the study intervention for those where audit data was being reported

## Conclusions

The most important finding of this systematic literature review is as follows: the sole use of face-to-face interaction (as an implementation strategy) between pharmacists and other healthcare professionals (e.g. as part of a multi-disciplinary team on a ward) has been shown to consistently and significantly improve the process outcomes (e.g. rate of screening for a comprehensive set of cardiometabolic risk factors or metabolic syndrome) for those with severe mental illness. Implementation strategies which did not include any form of face-to-face contact appear to be less effective for process outcomes. Despite being frequently employed within studies, the sole use of reminders (e.g. pop-up alerts on computer systems) appears to have no statistically significant impact on process outcomes. We would recommend the incorporation of face-to-face interaction as part of any implementation strategy chosen and discourage the sole dependence on pop-up alerts.

There is paucity of good quality qualitative and mixed methods design studies which include clinical outcomes and the association between specific process outcomes and improvements in clinical outcomes and also studies conducted in primary care with community pharmacy teams. Qualitative data will provide important information about the views, experiences and perceptions of key stakeholders (e.g. patients, informal carers and care professionals) about pharmacy. This type of data will inform current and future practice as well as other qualitative and quantitative research studies.

Mixed method studies would be instrumental in the development and testing of interventions delivered by pharmacy—in the development of the intervention, during the evaluation of the intervention, and after the follow-up and assessment of outcomes is completed. Mixed methods study designs also mitigate some of the intrinsic weakness or intrinsic biases and the problems that come from single method studies. Studies conducted in primary care and community are vital as there is great potential for impact; a significant proportion of people with mental health problems are cared for entirely within primary care and a significant proportion of the population can access a community pharmacy a short walk from where they live.

## Supplementary Information

**Additional file 1.** PRISMA-2009-Checklist-MS-Word.

**Additional file 2.** Supplementary material relating to systematic review and quality assessment.

**Additional file 3.** Effective Practice and Organisation of Care (EPOC) Taxonomy of interventions.

## Data Availability

All data used in this review are available in the included primary studies.

## Abbreviations

SMI: Severe Mental Illness
CVD: Cardiovascular Disease
CMR: Cardiometabolic Risk
MetS: Metabolic Syndrome
RCT: Randomised Controlled Trial
EPOC: Effective Practice and Organisation of Care
GP: General Practitioner
HbA1c: Glycosylated Haemoglobin
PRISMA: Preferred Reporting Items for Systematic Reviews and Meta-Analyses
BMI: Body Mass Index
MMAT: Mixed Methods Appraisal Tool
