# Supplementary material for: The role of pharmacy in the management of cardiometabolic risk, metabolic syndrome and related diseases in severe mental illness: a mixed-methods systematic literature review
Source: Syst Rev. 2021 Mar 31;10:92. doi: 10.1186/s13643-021-01586-9 (PMC8015120; doi:10.1186/s13643-021-01586-9)
Supplement: Supplementary file 2 — Additional file 2. Supplementary material relating to systematic review and quality assessment. [file 13643_2021_1586_MOESM2_ESM.docx]

**Additional file 2:**

**Supplementary material relating to systematic literature review and quality assessment**

(Please note that the references for this additional file appear at the end of this file)

- 1. **Review search strategy**

***Scoping search***

The Centre for Reviews and Dissemination (CRD) database and the Cochrane Database of Systematic Reviews library were initially searched. This was done in order to identify any ongoing or previously published systematic reviews of the role of pharmacy in the management of cardiometabolic risk (CMR), metabolic syndrome (MetS) or related diseases in severe mental illness (SMI).

DS conducted preliminary scoping searches for relevant studies within medical and social science databases (Prospero, Cochrane library, NICE Evidence Search, Turning Research Into Practice) using the following terms:

‘pharmac*’;

AND ‘mental’ OR ‘psych’;

AND ‘lifestyle advice’ OR diet OR smoking OR alcohol OR exercise OR metabolic OR diabetes.

Using these terms was, however, problematic as the number of results returned was very large and included many papers that were not specific to the question. The reason for this is that the term pharmac* included other areas such as pharmacological and pharmaceutical. The search was repeated to include more specific terms: pharmacy and pharmacist. This returned a smaller number of more specific and relevant papers (1–3).

The scoping searches identified that the literature exploring the role of pharmacy role of pharmacy in the management of cardiometabolic risk (CMR), metabolic syndrome (MetS) or related diseases in severe mental illness (SMI; therefore the search was expanded to include any pharmacy related activities by using a broader search terms in accordance with guidance from the CRD (4) and the Cochrane Handbook for Systematic Reviews of Interventions (5). The PICOS (Population, Intervention, Comparators, Outcome, Study design) tool (6) was used to inform the development of inclusion and exclusion criteria and search terms and identify search concepts for the literature search.

***Final search strategy***

We conducted a systematic search for primary research studies in which the study intervention involved pharmacy/pharmacy staff for CMR or MetS or related diseases in severe mental illness (SMI). We included any published literature which described an intervention involving pharmacy or pharmacy staff in CMR, MetS or related diseases were included (in all cases we describe this as the study intervention in our review), this could have included for example new pharmacy service or evaluations of existing services. Eleven electronic databases were searched from inception to January 2018; Medline, EMBASE, PsycINFO, British Nursing Index, AMED, Health Business Elite, Health management information consortium, The Cochrane Library, Health Technology Assessments, Scopus, and Web of Science. Database-specific search strategies were developed with the input from a medical librarian. Search terms included a combination of Medical Subject Heading terms, keywords and a comprehensive list of synonyms of the following: “pharmacist” OR “pharmacy” AND “cardiometabolic” OR “ metabolic syndrome” and “severe mental illness” with the aim of being as sensitive as possible. The search was not limited by dates of publication or country of origin. The database search was supplemented by search of the Ethos repository of theses; hand-searches of key journals and conference proceedings; citation searches of highly cited key studies; scanning reference lists of key studies; contacting authors (once) of relevant conference abstracts and studies and experts in the field. The grey literature search was further supplemented by checking the first 100 hits from Google Scholar, the Grey Literature in Europe website (http://www.opengrey.eu/) and by consulting relevant agencies or organisations. The reference manager tool used (Mendeley) also provides a service known as Mendeley suggest which provided a further source of possible studies.

***Search terms***

Preliminary search terms were generated after scoping reviews and consultation with academic supervisors and the medical librarian. Evaluation was based on whether these search terms provided comprehensive searches which would generate relevant articles. This allowed the search terms to be developed and refined through an iterative process.

The final search terms were:

1. Pharmacy OR pharmacist OR “pharmacy education” OR “pharmacist education” OR “pharmaceutical care”
2. Mental OR psych*
3. Screening OR monitoring” OR Intervention
4. life-style OR “public health” OR health promo*
5. Obesity OR overweight OR weight OR BMI or “body mass index” OR “weight management” OR nutrition OR “weight loss” OR eating OR diet*
6. Cardio* OR cardiac
7. Metabolic OR cardiometabolic OR cardio* OR “syndrome X” OR “cholesterol” OR “triglycerides” OR lipid*
8. Diab* OR glucose OR “glucose regulation” OR “glucose intolerance” OR “hyperglycaemia” or “HbA1c”
9. Exercise OR “physical activity” OR walk* OR jog* OR run*
10. Smoking OR “smoking cessation” OR “nicotine replacement" OR “quit* smoking”
11. Alcohol OR drink* OR “problem drinking”
12. Hypertension OR “blood pressure” OR “BP”
13. 1 AND 2 AND 3 AND 4
14. 1 AND 2 AND 3 AND 5
15. 1 AND 2 AND 3 AND 6
16. 1 AND 2 AND 3 AND 7
17. 1 AND 2 AND 3 AND 8
18. 1 AND 2 AND 3 AND 9
19. 1 AND 2 AND 3 AND 10
20. 1 AND 2 AND 3 AND 11
21. 1 AND 2 AND 3 AND 12

***Inclusion and exclusion criteria***

The inclusion and exclusion criteria, developed from the scoping searches, for the review are outlined in the table below.

**Table 1.1 PICOS Criteria** (6)**.**

| Category | Inclusion Criteria | Exclusion Criteria |
| --- | --- | --- |
| Participants | Individuals with severe mental illness (SMI) ≥ 18 years.  Severe mental illness – bipolar affective disorder, schizophrenia, schizoaffective disorder, psychosis, any psychotic disorder. | Individuals with SMI under the age of 18 years. |
| Intervention | Pharmacy staff carrying out any of the following activities to any degree:  Screening/monitoring for cardiometabolic risk or metabolic syndrome, syndrome X or cardiometabolic disease or related diseases and any of the associated risk factors including lifestyle advice, diet, smoking, alcohol, exercise, cardiovascular disease, diabetes or prediabetes,HbA1c, glucose, weight, BMI, waist circumference, overweight, obesity, lipids, lipid abnormalities, blood pressure and hypertension.  Health promotion or risk reduction intervention for cardiometabolic risk or metabolic syndrome, syndrome X or cardiometabolic disease or related diseases or any of the associated risk factors.  Medicines management activities relating to the above. | Any of these activities carried out wholly by staff who are not pharmacy staff.  Activities that are carried out by pharmacy staff who are not listed. |
| Comparators (NB it is not compulsory to have a comparator for the study to be included) | Patients with SMI ≥ 18 years who did not receive any intervention.  Patients who don’t have SMI who have any intervention. |  |
| Outcome | Primary outcome:  Change in rate of screening of cardiometabolic risk or metabolic syndrome, cardiometabolic diseases or syndrome X or any of the associated risk factors.  Change in health behaviour (risk reduction or health promotion).  Diagnosis of metabolic syndrome or identification of individual at high risk of metabolic syndrome.  Diagnosis of diseases related to cardiometabolic risk metabolic syndrome including diabetes, cardiovascular disease, hypertension, obesity, overweight, diabetes/high risk of diabetes/pre-diabetes.  Change in patient or physical health parameter e.g. BP outcome for the above.  Views, perception, opinions, experiences of service users, carers or any care professionals on the role of pharmacy to deliver ANY of the interventions. | Studies that do not measure the primary outcomes.  . |
| Study Design | Any study design.  Any country.  Papers written in English only. | No study design will be excluded.  Papers not written in the English language. |

***Participants***

Individuals with SMI who were aged 18 or over were included in the review. Studies were excluded if they did not include an explicit reference to these participants. There were no limits set on study participants in terms of study setting (i.e. hospital, community or academic institution) or gender. Studies conducted within the UK, European and international settings were included.

***Interventions***

To be included the study intervention involved pharmacy or pharmacy staff in CMR or MetS or related diseases in severe mental illness. We included any published literature which described an intervention involving pharmacy or pharmacy staff in CMR, MetS or related diseases were included this could include for example a new pharmacy service or an existing service. This could include, for example, pharmacists/pharmacy staff/pharmacy students/pharmacy service for undertaking screening (e.g. weight checks), identifying abnormality (e.g. metabolic syndrome), managing cardiometabolic risk factors (e.g. providing support for smoking cessation) or advising on medication (e.g. advising on switching medication with lower risk profile for weight gain). In all cases we describe this as the study intervention in our review. The intervention should be for those ≥ 18 years with SMI could be for CMR risk factors or MetS or related diseases as a whole or its constituent parts or specific lifestyle behaviours.

***Comparators***

Initial searching revealed that studies which included a comparison group appeared to be small in this research area, therefore studies with and without a comparison group were included in the review.

***Outcomes***

The primary outcome measures were change in rate of screening of cardiometabolic risk factors metabolic syndrome ore related diseases or any of the associated risk factors including lifestyle advice, diet, smoking, exercise, cardiovascular disease; change in health behaviour (risk reduction or health promotion); diagnosis of metabolic syndrome or identification of individual at high risk of metabolic syndrome; diagnosis of diseases related to metabolic syndrome including diabetes, cardiovascular disease, hypertension, obesity, overweight, diabetes/high risk of diabetes or change in patient outcome or health parameter e.g. BP for the above.

***Study Design***

The decision was made to include mixed methods, qualitative and quantitative study designs in the current review. In recent times there has been increasing recognition of the value of qualitative evidence when assessing interventions (7,8). In addition what has become more common health intervention evaluation is the inclusion of qualitative aspects and for the evaluation of complex interventions a ‘mixed methods’ approach (9,10). In the understanding “*what, how and why*” qualitative evidence is very valuable in reviews of intervention effectiveness (11). Qualitative research data help in the interpretation of systematic review as it enhances the understanding of the what parts are seen favourably/unfavourably and how those who receive the intervention experience it (5).

***Language and year of publication***

Non English-language papers found during the search process were not included for final review as there were no resources to translate. This was documented along with the reason for exclusion. No limitations on the year of publication were applied

**1.2 Reasons for excluding studies after full text review**

**Table 1.2 Reasons for excluding studies after full text review**

| # | Study Name | Reason for Exclusion |
| --- | --- | --- |
|  | Alderman CP, Lucca JM. Psychiatry and clinical pharmacy: A logical partnership. Indian Journal of Psychiatry. 2017;59(2):138-140. | Editorial, not a study of an intervention. |
|  | Attard A, McRobbie D, Taylor DM, West T. A career as ... a psychiatric liaison pharmacist. Hospital Pharmacist. 2008;15(3):99-100. | General overview of a career role – not a study of an intervention. |
|  | Augsten A, Correa-Vento M, Cousins S, Bober D, Camejo M, Levy B, Tucker T. Long-acting therapy clinic: A pharmacy initiative for the treatment of serious mental illness. Journal of Pharmacy Practice. 2016;29(3):331. | No outcomes measured or reported. |
|  | Ball MP, Hooper ET, Skipwith DF, Cates, ME. Clozapine-induced hyperlipidemia resolved after switch to aripiprazole therapy. Annals of Pharmacotherapy. 2005;39( 9):1570-2. | No mention of pharmacist/pharmacy |
|  | Baudry S. Four years evaluation on schizophrenic patients who followed a therapeutic education program called drug's workshops. European Archives of Psychiatry and Clinical Neuroscience. 2010;260. | No mention of cardiometabolic risk factors or metabolic syndrome or related diseases |
|  | Bell JS; Whitehead P, Aslani P, McLachlan AJ, Chen TF. Drug-related problems in the community setting: Pharmacists' findings and recommendations for people with mental illnesses. Clinical Drug Investigation. 2006;26(7):415-25. | Nothing specific for cardiometabolic risk factors or metabolic syndrome or related diseases. Diagnoses of participants not reported. |
|  | Binford SH, Johnson MD, Kennedy RS, Leffler JB. Clinician education improves lipid monitoring in patients taking second-generation antipsychotic agents, nationally and locally. Health Outcomes Research in Medicine. 2012;3(3). | Pharmacy/pharmacist not involved. |
|  | Bradley BA. Psychiatric pharmacist in a federally qualified health center primary care clinic. Journal of Pharmacy Practice. 2013;26(3):341-2. | No explicit mention of cardiometabolic risk factors or metabolic syndrome or related diseases. Diagnoses of individuals on antipsychotics not stated. |
|  | Brunette MF, Dzebisashvili N, Xie H, Akerman S, Ferron JC, Bartels S. Expanding cessation pharmacotherapy via videoconference educational outreach to prescribers. Nicotine and Tobacco Research. 2015;17(8):960-7. | Pharmacy/pharmacist not involved. |
|  | Butler P, Simonson C, Goldie C, Kennedy A, Goldstone L. Baseline metabolic monitoring of atypical antipsychotics in an inpatient setting. Mental Health Clinician. 2013;3(3):122-8.  And  Butler P, Goldie C, Simonson C, Goldstone L, Kennedy A. Inpatient Pharmacist Intervention Helps Sustain Improved Rates of Baseline Metabolic Monitoring for Patients Initiated on Atypical Antipsychotics.  <http://www.pharmacy.arizona.edu/sites/default/files/Abstracts%20for%20Senior%20Projects%202014.pdf>  (second reference discussing outcomes of the first) | First paper describes the process the second paper describes the outcomes. Diagnoses of participants not stated. |
|  | Buxton JA, Chandler-Altendorf A, Puente AE. A novel collaborative practice model for treatment of mental illness in indigent and uninsured patients. American Journal of Health-System Pharmacy. 2012;69(12);1054-62. | No mention of cardiometabolic risk factors or metabolic syndrome or related diseases |
|  | Caballero J, Souffrant G, Heffernan E. Development and outcomes of a psychiatric pharmacy clinic for indigent patients. American Journal of Health-System Pharmacy. 2008;65(3):229-33. | Diagnoses of participants not stated. |
|  | Canales PL, Dorson PG, Crismon ML. Outcomes assessment of clinical pharmacy services in a psychiatric inpatient setting. American Journal Health System Pharmacy. 2001;58(14):1309-16. | No mention of cardiometabolic risk factors or metabolic syndrome or related diseases. Diagnoses of participants not stated. |
|  | Canning JE, Nelson LA, Elliott E, Hieber R, Liu Y. Assessment of adherence with metabolic monitoring before and after implementation of a pharmacy-driven computerized metabolic monitoring database. Journal of Pharmacy Practice. 2011;24(2);264-5. | Conference abstract. No outcomes reported. Diagnoses of participants not reported. |
|  | Christopher M, Popish S, Yee M, Furman A, Kimura H. Academic detailing engages members of the healthcare team and veterans in improving care for mental health conditions. 2012;25(2):293. | Conference abstract. No mention of cardiometabolic risk factors or metabolic syndrome or related diseases |
|  | Chung B, Dopheide J, Gregerson P. Psychiatric Pharmacist and Primary Care Collaboration at a Skid-Row Safety-Net Clinic. Journal of the National Medical Association. 2011;103(7):567-74. | Nothing specific relating to cardiometabolic risk factors or metabolic syndrome or related diseases |
|  | Cobb CD. Optimizing medication use with a pharmacist-provided comprehensive medication management service for patients with psychiatric disorders. Pharmacotherapy. 2014;34(12):1336-40. | Outcomes related to cardiometabolic monitoring or metabolic syndrome or related diseases not reported. |
|  | DeJongh B, Garcia G, Parra D, Fernandez-Milo A.. Design and implementation of a clinical pharmacist managed metabolic syndrome clinic in a mental health setting. Journal of Pharmacy Practice. 2011;24(2):268. | Work in progress – no outcomes reported. Diagnoses of participants not reported. |
|  | Dhamane AD, Hudson TJ, Martin BC, Said Q, Brixner DI. Metabolic monitoring of patients prescribed second-generation antipsychotics. Journal of Psychiatric Practice. 2013;19(5):360-74. | No pharmacist involved. No intervention implemented. Diagnoses of participants not reported. |
|  | Dhamane AD, Martin B, Hudson TJ, Said Q, Brixner D. Assessment of metabolic monitoring of patients prescribed second generation antipsychotics (SGAs) using electronic medical record (EMR) data.  Value in Health. 2010;13:A99. | Poster/conference abstract. No pharmacist involved. No intervention implemented. Diagnoses of participants not reported. |
|  | Dopheide JA, Bostwick JR, Goldstone LW, Thomas K, Nemire R, Gable KN, Cates M, Caballero J, Smith T, Bainbridge J. Curriculum in psychiatry and neurology for pharmacy programs. American Journal of Pharmaceutical Education.2017;81(7). | Focused on educational programs of pharmacy professionals nothing specific to severe mental illness or metabolic or cardiometabolic risk factors or related diseases. Not a study of an intervention. |
|  | Dorman C, Winkler H, Moore TA. Implementation and outcomes of mental health pharmacist-managed electronic consults at a VA health system. Journal of pharmacy practice. 2016;29(3):311. | Nothing specific relating to cardiometabolic risk factors or metabolic syndrome or related diseases. |
|  | Edelsohn GA, Parthasarathy M, Terhorst L, Karpov IO, Schuster J. Measurement of metabolic monitoring in youth and adult medicaid recipients prescribed antipsychotics. Journal of Managed Care Pharmacy. 2015;21(9):769-77. | Not a study of an intervention. |
|  | Erickson ZD, Kwan CL; Gelberg HA; Arnold IY; Chamberlin V; Rosen JA, Shah C, Nguyen CT, Hellemann G, Aragaki DR, Kunkel CF, Lewis MM, Sachinvala N, Sonza PA, Pierre JM, Ames D. A Randomized, Controlled Multisite Study of Behavioral Interventions for Veterans with Mental Illness and Antipsychotic Medication-Associated Obesity. Journal of General Internal Medicine. 2017;32:32-9. | Pharmacy/pharmacist not involved in the intervention. |
|  | Gallimore CE, Sokhal D, Zeidler Schreiter E, Margolis AR. Pharmacist medication reviews to improve safety monitoring in primary care patients.  Families, Systems and Health.2016;34(2):104-13. | Diagnoses of participants not reported. No mention of cardiometabolic risk factors or metabolic syndrome or related diseases. |
|  | Ganzer N, Utter B, Dejongh B, Behrens M, Garcia G, Graham R. Re-implementation of a pharmacist-managed metabolic syndrome clinic in an outpatient mental health clinic setting. Mental health clinician. 2015;5(1):57-62. | Diagnoses of participants not reported. |
|  | Gavin L, Frey T. Assessment of a pharmacist-run medication education group for inpatient psychiatric patients. Mental Health Clinician. 2012;2(4):94-9. | No mention of cardiometabolic risk factors or metabolic syndrome or related diseases. Diagnoses of participants not reported. |
|  | Gisev N, Bell JS, O'Reilly C, Rosen A, Chen TF. An expert panel assessment of comprehensive medication reviews for clients of community mental health teams. Social Psychiatry and Psychiatric Epidemiology. 2010;45(11):1071-9. | Reported outcomes are not explicit enough to examine specific data for cardiometabolic risk factors or metabolic syndrome or related diseases |
|  | Green S, Beveridge E, Evans L, Trite J, Jayacodi S, Evered R, Parker C, Polledri L, Tabb E, Green J, Manickam A, Williams J,Deere R, Tiplady B. Implementing guidelines on physical health in the acute mental health setting: a quality improvement approach. Interntional Journal Mental Health Systems. 2018;12:1. | Diagnoses of participants not reported. |
|  | Greg Deardoff O, Nelson L, Elliott E, Liu Y, Schmidt S Stoner S, Nelson LA, Liu Y. Adherence and clinical outcome of metabolic monitoring in psychiatric patients transitioning from an inpatient to outpatient setting. Journal of Pharmacy Practice. 2011;24(2):263. | Work in progress. Conference poster abstract. No outcomes reported. No pharmacy/pharmacist intervention. |
|  | Griebe KM, Caniff KE, Bostwick JR. Psychiatric Pharmacists: Key Allies to Improve Antipsychotic Metabolic Monitoring. Psychiatric Services. 2016;67(4):469-70. | General discussion of the topic not a study of an intervention |
|  | Hammad EA, Yasein N, Tahaineh L, Albsoul-Younes AM. A randomized controlled trial to assess pharmacist- physician collaborative practice in the management of metabolic syndrome in a University medical clinic in Jordan. Journal of Managed Care Pharmacy. 2011;17(4):295-303. | Diagnoses of participants not stated. |
|  | Hannou S, Pannatier A, Von Gunten A, Voirol P, Mall JF, De Giorgi I, De Boer E. CP-076 Effects of pharmacist interventions on inappropriate prescribing in a geriatric psychiatry unit. European Journal of Hospital Pharmacy: Science and Practice. 2014;21:A31. | Interventions carried out by pharmacist did not relate to cardiometabolic risk factors or metabolic syndrome or related diseases. Diagnoses of participants not reported. |
|  | Hattingh HL, Scahill S, Fowler JL, Wheeler AJ. Exploring an increased role for Australian community pharmacy in mental health professional service delivery: evaluation of the literature. Journal of Mental Health. 2016;25(6): 550-9. | This is a narrative literature review of systematic reviews. Not a study of an intervention. |
|  | Hor ES, Subramaniam S, Koay JM, Bharathy A, Vasudevan U, Panickulam JJ, Ng I, Arif NH, Russel V. Improving metabolic monitoring in patients maintained on antipsychotics in Penang, Malaysia. Australasian Psychiatry. 2016;24(1):,67 – 71. | No pharmacy/pharmacist involved. |
|  | Huynh P, Winkler H, Moore TD, Nelson J. Primary care mental health integration: Evaluating the impact of behavioral health clinical pharmacy specialists. Journal of Pharmacy Practice. 2015;28(3):354. | Work in progress. Conference abstract. Not severe mental illness (anxiety, depression and post-traumatic stress disorder). Not cardiometabolic risk factors or metabolic syndrome or related diseases. |
|  | Ishkova-Volovets N. Improving adherence to metabolic monitoring guidelines for second-generation antipsychotics: The College of St. Scholastica; 2014. | Thesis for doctor of nursing practice. Pharmacy/pharmacist not included. |
|  | Johnson K, Frede S, Fuller L, Weed E, Luder H. Identification and management of metabolic and movement abnormalities in patients taking antipsychotic medications in a community pharmacy. 2016; 56(3). | Conference abstract. No outcomes reported. Diagnoses of participants not reported. |
|  | Kamath J, Singh R, Chen X, Wu H. Implementation of metabolic monitoring guidelines for patients receiving antipsychotic medications in a large outpatient psychiatry clinic: Interventions and outcomes. Neuropsychopharmacology. 2013;38:S517-8. | No information about diagnoses of participants. Pharmacist/pharmacy not involved. |
|  | Kim S. Clozapine support group therapy provided by the medication therapy management pharmacist from university outpatient psychosis treatment program. Journal of the American Pharmacists Association. 2013;53(2). | Conference abstract. Outcomes not reported clearly. Diagnoses of participants not reported. |
|  | Kim S. Smoking cessation support group under pharmacist auspice at the university outpatient psychiatric clinic. Journal of Pharmacy Practice. 2016;29(3):333. | Conference abstract. Diagnoses of participants not reported. |
|  | Klein M, Mahoney L, Kruse K, Pinter M. Impact on monitoring of fasting lipid panel and fasting plasma glucose in patients prescribed second generation antipsychotics with the implementation of an ambulatory pharmaceutical care service. 2010;23(2):169. | Conference abstract. Diagnoses of participants not reported. |
|  | Kroening J. Community based psychiatric outpatient metabolic monitoring for atypical antipsychotics. The College of St. Scholastica; 2016. | Thesis for doctor of nursing practice. Pharmacy/pharmacist not included. |
|  | Kurian J, Ramesh M, Rajesh, R, Kishore, M. Impact of collaborative coustomized patient education in psychiatric diseases. Value in health. 2017;20(5). | Conference abstract. Nothing related to metabolic syndrome or cardiometabolic risk factors or related diseases. |
|  | Lai CL, Chan HY, Pan YJ, Chen CH. The effectiveness of a computer reminder system for laboratory monitoring of metabolic syndrome in schizophrenic outpatients using second-generation antipsychotics. Pharmacopsychiatry. 2015 ;48(1):25-9. | No pharmacy/pharmacist involvement. |
|  | Li L.,Kirwin PD, Rosenheck R. Multimorbidities: Age-related relationships among medical disorders, psychiatric disorders, substance use disorders, and psychotropic prescriptions in the U.S. Veteran population. American Journal of Geriatric Psychiatry. 2016;24(3). | AGM conference abstract. No pharmacy/pharmacists involvement or intervention. |
|  | Maslen CL, Rees L, Redfern PH. Role of the community pharmacist in the care of patients with chronic schizophrenia in the community. Journal of Pharmacy Practice. 1996;4(4):187-95. | Questionnaire study understanding the attitudes of community pharmacists to schizophrenia, but nothing related to metabolic syndrome/cardiometabolic risk factors or related diseases. |
|  | Maulavizada H, Emmerton L, Laetitia HL. Can a pharmacy intervention improve the metabolic risks of mental health patients? Evaluation of a novel collaborative service. BMC Health Services Research. 2016;16(1):146 | Diagnoses of participants not reported. |
|  | McCleeary-Monthei E, Kutscher EC. Evaluation of lipid and glucose monitoring after implementation of a pharmacist initiated antipsychotic monitoring form. Journal of Pharmacy Practice. 2012;25(2);281. | Conference abstract. Work in progress. Diagnoses of participants not reported. No outcomes reported. |
|  | McCue , Lacro JP. Effect of clinical pharmacist involvement with the mental health intensive case management (MHICM) team at VA San Diego healthcare system. Journal of Pharmacy Practice. 2011;24(2):247. | Conference abstract. Outcomes of monitoring not reported clearly. |
|  | Moeller KE, Rigler SK, Mayora A, Nazir N, Shiremand TI. Quality of monitoring for metabolic effects associated with second generation antipsychotics in patients with schizophrenia on public insurance. Schizophrenia Research. 2011;126(1-3):117-23. | Pharmacy/pharmacist not involved. |
|  | Montgomery J, Turner TJ, Quinn M, Harms J, Samol S, Fabian T. The role of a community pharmacy resident in expanding clinical pharmacy services within a traditional dispensing model. Pharmacotherapy. 2011;31(10). | Diagnoses of participant not reported. Outcomes not reported in detail. |
|  | Morrato EH, Campagna EJ, Brewer SE. Programmatic efficiency of a pharmaceutical risk management program: Outcomes from diabetes screening of adults receiving antipsychotics. Pharmacoepidemiology and Drug Safety.2016;25:310-1. | Not a study of a pharmacy/pharmacist intervention. It is a calculation checking to assess screening yield and number needed to screen to detect new cases of diabetes of individuals on antipsychotics. |
|  | Nelson LA, Graham MR, Lindsey CC, Rasu RS. Medication adherence, lipid and glycemic control in patients with psychotic disorders compared to patients without psychiatric illness receiving care in a veterans affairs medical system. Journal of Pharmacy Practice. 2011;24(2):248. | Not a study of an intervention. |
|  | Onatade R, Oduniyi O. Improving the pharmaceutical care of patients on psychotropic medication admitted to an acute hospital – the impact of a proactive ‘inreach’ specialist psychiatric pharmacist service. Poster presented at United Kingdom Clinical Pharmacists Association conference 2016. | Unclear as to whether the diagnoses of the participants includes severe mental illness or not. |
|  | Owen RR, Drummond KL, Viverito KM, Marchant K, Pope SK, Smith JL, Landes RD. Monitoring and managing metabolic effects of antipsychotics: a cluster randomized trial of an intervention combining evidence-based quality improvement and external facilitation. Implementation science. 2013;8:120. | No pharmacy/pharmacist involvement. Diagnoses of target population for intervention not stated. |
|  | Parker S, Henderson R, Dutton T, Pate JR, Bean JR. Metabolic monitoring for patients on second-generation antipsychotics using electronic notifications as a reminder system for providers: 266. Pharmacotherapy. 2016; 36(12):e272. | Diagnoses of participants not reported. Not clear if intervention implemented by pharmacy/pharmacist. |
|  | Peh AL. Safety monitoring of patients on atypical antipsychotics.  Source Quality & safety in health care. 2008;17(6):469-72. | Diagnoses of participants not reported. |
|  | Pringsheim T, Kelly M, Urness D, Teehan M, Ismail Z, Gardner D. Physical Health and Drug Safety in Individuals with Schizophrenia. 2017;62(9);673-83. | Review of National Institute for Health and Care Excellence and Scottish Intercollegiate Guidelines Network guidelines. Not a study of an intervention. |
|  | Raphael C, Fedoruk E. Practice spotlight: Pharmacists of the Centre for Addiction and Mental Health. Canadian Journal of Hospital Pharmacy. 2008;61(5:)346-7. | Discussion of service; not a study of an intervention, no outcomes reported. |
|  | Riesselman A, Baker JM, Bodenberg M,Lucas P,Strobl B. Metabolic syndrome monitoring program: A clinical program to screen patients for metabolic syndrome in a state psychiatric hospital. Journal of Pharmacy Practice. 2011;24(2):275-6. | Work in progress. Conference poster abstract. No outcomes reported, Diagnoses of participants not reported. |
|  | Roestenburg S, Brewster M, Stock CJ, LaFleur J. Clinical outcomes of veterans with serious mental illnesses enrolled in a pharmacist-managed metabolic monitoring clinic: A retrospective cohort study. Journal of Pharmacy Practice. 2015;28(3):338-9. | Conference abstract. Outcomes of intervention not reported. |
|  | Sano T, Inoue M, Takizawa M, Shimamori Y, Kurosawa N. The Effect of Pharmacist-led Psychiatric Pharmacotherapy Conferences on the Appropriate Use of Antipsychotics. Journal of the Pharmaceutical Society of Japan. 2017;137(5):603-10. | Not available in English. |
|  | Schellack N, Matlala M. Providing an overview of antipsychotic drugs: Is schizophrenia a psychiatric challenge? Pharmaceutical Journal. 2014;81(4):28-33. | Review article. Not a study of an intervention. |
|  | Schmitz A, Rohrich M, Newman W, Wolf P. Cardiometabolic management in severe mental illness requiring an atypical antipsychotic. Mental Health Clinician. 2017;7(2):81-7. | Not a study of a pharmaceutical/pharmacy intervention. |
|  | Seng KH. Reducing polypharmacy for elderly psychiatric patients for safe and high quality care. Quality and Safety in Health Care. 2009;18(4). | Conference abstract. No outcomes reported. |
|  | Shishko I, Oliveira R, Moore TA, Smith AG. Pharmacist driven metabolic monitoring clinic of atypical anti psychotics. Journal of Pharmacy Practice; 2016;29(3):318-9. | Conference abstract. Work in progress. No outcomes reported. Diagnoses of participants reported. |
|  | Stutzman D, Reta A, O'Callaghan R. Psychotropic prescribing practices and physician attitudes on integrating psychiatric pharmacist services in an ambulatory care setting. Journal of Pharmacy Practice. 2015;28(3):355. | Conference abstract. Work in progress. No outcomes reported. Diagnoses of participants not stated. |
|  | Su HC, Chen CH, Chan AL. Cost of pharmaceutical care in patients with metabolic syndromes caused by atypical antipsychotics. Value in Health. 2010;13(3). | Diagnoses of participants not reported. |
|  | Tai M, Lee B, Onukwugha E, Zito JM, Reeves GM, dosReis S. Impact of Coordinated Behavioral Health Management on Quality Measures of Antipsychotic Use. 2017. | Not a study of an intervention. Quasi-experimental design to evaluate the average treatment effect of the care coordination using a wraparound practice model on improving the quality of antipsychotic use. |
|  | Tatreau JR, Harris S, Sheitman B, Steiner BD. Cardiometabolic Assessment, Diagnosis, and Treatment of Chronic Medical Illnesses During an Inpatient Psychiatric Hospitalization: Colocated Medical Care Versus Treatment as Usual. The primary care companion for CNS disorders. 2016;18(6). | Not a study of an intervention implemented by pharmacist; no pharmacy/pharmacist involvement. |
|  | Traynor K. CMS paves way for pharmacists to help blunt metabolic effects of antipsychotics. 2017;74(14):1037-8. | Reporting on the work of another pharmacist, diagnoses of participants not reported. |
|  | Wang I, Dopheide JA, Gregerson P. Role of a psychiatric pharmacist in a Los Angeles "Skid-Row" safety-net clinic. Journal of urban health: bulletin of the New York Academy of Medicine. 2011;88(4):718-23. | Interventions for metabolic syndrome or cardiometabolic risk factors or related diseases not explicitly stated. Unable to discern outcomes from the results reported. |
|  | Ward K, Samara W, Popish S, Furman A, Meier J. Use of a clinical dashboard to improve cardiometabolic syndrome monitoring. Journal of Pharmacy Practice. 2012;25(2):297. | Diagnoses of participants not reported. Outcomes not reported. |
|  | Wenger PJ, Mays KR. Effect of pharmacy team interventions on monitoring rates for second-generation antipsychotics in a correctional setting. Pharmacotherapy. 2012;32 (no. 10). | Diagnoses of participants not reported. |
|  | Wiechers IR. Improving psychopharmacological care for older veterans: Implementation of phase 2 of the psychotropic drug safety initiative. Journal of Geriatric Psychiatry. 2016;24(3). | Conference abstract. No outcomes reported. Diagnoses of participants not reported. |
|  | Williams T, Purvis TL. Development of an outpatient pharmacist-managed clozapine clinic. American journal of health-system pharmacy. 2012;69(14):1192-5. | Outcomes not explicitly reported. |
|  | Wilson E, Randall C, Patterson S, Emmerson B, Moudgil V, Weaver T. Monitoring and management of metabolic abnormalities: mixed-method evaluation of a successful intervention. Australasian psychiatry. 2014;22(3):248-53. | Pharmacy/pharmacist not involved. Diagnoses of participants not reported |
|  | Xiangjun K. To ensure all ward 22a patients on atypical antipsychotics are monitored according to protocol. Annals of the Academy of Medicine Singapore. 2012;41(9). | Diagnoses of participants not reported. |
|  | Zekovic A, Kristensen S, Pedersen LL. Implementation of clinical pharmacy in the acute psychiatric wards: Improving quality of medical treatment across health care sectors. International Journal of Clinical Pharmacy. 2017;39(1):228. | Oral symposium abstract. Diagnoses of participants not reported. No outcomes explicitly reported. |

- 1. **Quality assessment**
     1. **Quality assessment – details regarding the mixed methods appraisal tool (MMAT)**

The methodological quality of the selected studies was assessed to identify weaknesses and strengths in order to help interpretation of results. Papers were quality assessed using the Mixed Methods Appraisal Tool (MMAT) (12,13) which is designed to appraise the methodological quality of qualitative, mixed methods and quantitative literature. The MMAT has been shown to have high validity (14)and reliability (15). The scale comprises two screening questions, followed by individual items for different methodologies. Four domains: sampling strategy, appropriate measurement, sample representation and acceptable response rates for the chosen research tool (i.e. questionnaire) were used to assess quantitative studies. Qualitative studies were assessed according to: relevance of data source (i.e. focus groups), consideration of how findings relate to the context (i.e. setting), appropriateness of data analysis process (i.e. suitable information provided) and consideration of how the researcher influenced findings (i.e. interaction with participants). Studies were given an overall quality score for each domain met using the following star ratings: Four* = 100%, Three* = 75%, Two* = 50%, One* = 25%, No stars X = 0%

**MMAT screening questions:**

*1. ‘Are there clear qualitative and quantitative research questions (or objectives), or a clear mixed-methods question (or objective)?’*

*2. ‘Do the collected data allow for the research question (or objective) to be addressed?’*

**Quality assessment of studies for this review**

The MMAT was employed to review each of the 33 articles. However, two of the articles (16,17) were both from the same study and therefore assessed together as per the guidance of the author of this tool. As part of the review process, papers were divided into methodological domains: mixed methods, qualitative and quantitative. The quantitative domain is subdivided into three: randomised controlled, non-randomised and descriptive. The current review contains articles classified into five groups: qualitative studies (n=4), quantitative studies with a nonrandomised design (n=17), quantitative studies with a randomised control design (n=1), quantitative studies with a descriptive design (n=6), and mixed methods studies (n=4). The approach adopted to the classification of articles into these four groups was taken directly from the work on the MMAT as presented by Pluye et al, (12,13). The four articles classified as mixed-methods studies (18–21) did not conform to the classic mixed methods designs as described, for example, by Creswell and Plano Clark (22). The articles did not label themselves as being ‘mixed methods’, but they included qualitative and quantitative data which were collected and analysed with the purpose of meeting the overall research objective. In these cases the criteria for the appraisal of mixed-methods studies were applied. In accordance with guidance provided (13) those articles classified as mixed methods were awarded overall quality scores in line with the lowest score of their specific components.

14 of the studies in the review followed a pre-post interventional study design (1,2,31–34,23–30) and did not conform to the non-randomized control design as described in the MMAT tool as they did not include a control group. This issue is acknowledged by the author of the tool. They make the following recommendations based on other users’ experiences; ‘*remove item 3.3 on the comparability of groups, replace it with another item they judge important, interpret item 3.3 for single group pre-post by checking if the pre- and post-subjects are comparable (e.g., if a high drop-out rate at post) or use the descriptive set of criteria (since no comparison group is a common characteristic of descriptive studies). They go on to say that appraisal is a value judgment and the interpretation of the items might vary based on the context of the review (types of studies)’.*

After much discussion between the two of the authors (DS and EL) and conformation that this approach was acceptable with another author (RM) it was agreed to use a modified tool as follows:

*Original criteria:*

*‘3.2. Are measurements appropriate (clear origin, or validity known, or standard instrument; and absence of contamination between groups when appropriate) regarding the exposure/intervention and outcomes?’*

*‘3.3. In the groups being compared (exposed vs. non-exposed; with intervention vs. without; cases vs. controls), are the participants comparable, or do researchers take into account (control for) the difference between these groups?’*

*Modified criteria:*

*3.2. Are measurements appropriate (clear origin, or validity known, or standard instrument?*

*3.3. In the pre and post study groups are the participants comparable, or do researchers take into account (control for) the difference between or any contamination that may have occurred?*

There were several reasons for choosing to take this approach; removing the item altogether would have resulted in assessing those studies against three rather than four items leading to an inequity in the assessment if compared to assessment of those studies that did conform to the non-randomised control design. The question of contamination appeared to be unrelated to that of the measurement chosen. In addition including the question of contamination as a separate item is consistent with a previous version of the tool (14).

Three of the mixed methods studies (35–37) including many aspects that were not relevant to the review question and the quality assessment were only applied to those aspects that were relevant to the review question.

**1.3.2 Quality assessment – application of MMAT to chosen studies**

Table 2 provides detailed information. The overall quality of the reported studies, assessed using the Mixed Methods Appraisal Tool (MMAT) (12,13) generally good, with twelve studies scoring **** (100%), eight studies scoring *** (75%), and thirteen studies scoring ** (50%) or less.

One study (37) did not explicitly state a research question or objective, therefore we based our quality assessment upon whether the collected data answered the research questions of the present review. A common weakness across a number of studies concerned the use of qualitative data – authors were not clear about how this particular type of data answered the research question (18,19,37) Another common weakness was the lack of (18,19,37,38) or justification for method of synthesis (20); the quality of any study will be fundamentally undermined if an inappropriate choice or method of data collection or analysis is chosen.

Another common weakness was inadequate researcher reflexivity within both qualitative studies (35,37,38) and qualitative aspects of mixed methods studies (18–21) – there was no examination or critique of how the researcher impacted on the study or the participants.

Relevant national published clinical guidelines were used in several of the studies to allow for the identification of appropriate outcome measures (e.g. blood lipids, weight), however, in two studies where only a few of these outcome measures were chosen rather than all of those recommended this was not justified (20,25). An inadequate rate of outcome data (< 80%) was reported in several studies (2,3,39,40) only one of these accounted for this with high drop-out rate. In another study inadequate information was provided to allow for attrition to be calculated (26), thus compromising the objectivity of the evaluation of results by potentially introducing bias.

Thirteen of the studies included in this review were uncontrolled quasi (pre–post) studies. There is some evidence to suggest that this type of study may overestimate the effects of quality improvement interventions or intervention implementation (41)

We identified one randomised controlled trial in our study selection process (3) – in this study participants were unaware of their assignment group at the start of the study. However, as the trial intervention involved receipt of pharmacist input or no pharmacist input, it might be anticipated that the participant would become aware of the group they had been assigned to as time when on. In addition, the investigators were made aware of which study arm the participants had been randomised to. These factors introduce a high risk of bias into this study. In this same study (3) the outcome data was inadequate and the drop-out rate was high – so valid conclusions cannot be drawn from results obtained.

Threats to external validity compromise confidence in stating whether a particular study’s results are applicable outside of the study. The risk of selection bias was high in some of the studies (16,17,42–44) given the lack of randomisation and lack of control groups. For these studies, therefore, one cannot reliably determine whether the observed changes resulted from the intervention. A few studies utilised convenience sampling (16,17,42). The membership of a convenient population may indicate greater access to resources, better knowledge, social support, or even just geographic proximity to the researcher. The method of sampling in the study by Lizer was not clear (2). Five studies (18,42–45) lacked information about refusal, response rates or follow-up engagement.

Demographic data provides important information about research participants and are necessary to determine whether participants are representative of the target population. Demographic data was completely absent in three of the studies (23,34,45).

A threat to the internal validity of a study can occur if contamination occurs between the pre and post groups as was the case in several of the quasi studies (2,23,26,29,34) or differences between active and control groups are not accounted for. One study acknowledged the effect of contamination between groups (107) – (participants were admitted more than once during the study period) but did not account for it in the data analysis/synthesis. In addition, none of our included studies reported undertaking power analysis calculation to determine the minimum number of participants they required.

All four mixed methods studies (18–21) scored poorly (50% or less). These studies were not described by their authors as being ‘mixed methods’, but all included qualitative and quantitative data which were collected and analysed with the purpose of meeting the overall research objective. Of these only one (20) made reference to the use of mixed data being as being relevant to the research questions. Not unsurprisingly, therefore, all four mixed methods studies scored zero for integration of both qualitative and quantitative data as no integration was undertaken. In the absence of integration, the knowledge harvested is only equivalent to the sum of that derived from a qualitative study and a quantitative study undertaken separately, rather than achieving a “whole greater than the sum of the parts” (10). Despite these weaknesses, we believed that, overall, these studies were sufficiently robust to contribute to our review discussion.

**Table 1.3: Reviewed journal articles by MMAT quality criteria scores: first author (reference)**

| Score  Study  Type | 25% * | 50% ** | 75% *** | 100% **** |
| --- | --- | --- | --- | --- |
| Qualitative studies | Shanker (37) |  | McMorris (38)  Quirk (35) | Taylor (36) |
| Quantitative randomised controlled trials | Schneiderhan (3) |  |  |  |
| Quantitative non-randomized | Lizer (2) | McCleeary-Monthei (26)  Bozymski (40)  Pena (39) | Runcie (23)  DelMonte (25)  Sud (29) | Barnes (24)  Taveira (46)  Schneiderhan (1)  Kjeldsen (27)  Ramanuj (28)  Cohen (47)  Koffarnus (30)  Barnes (31)  Fischler (32)  Sasson (33) |
| Quantitative  descriptive  studies | MacHaffie (42) | Gable (43)  Raynsford (45) | Lucca (44)  Bozymski (48)  Sharma∆ (16,17) | Porras-Segovia (49) |
| Mixed  methods  studies | Ohlsen (18)  Watkins (19) | Lee (20)  Health foundation (21) |  |  |

∆both publications are from one study so assessed together as per guidance from authors of the MMAT tool.

**Detail of the screening questions, quality criteria and scoring of the articles included in the review**

**A: Application of MMAT criteria to qualitative studies:** **first author (reference) and score (indicated by number of stars)**

| Study    Question | Taylor (36)**** | McMorris (38) *** | Quirk (35)*** | Shanker (37)* |
| --- | --- | --- | --- | --- |
| Are there clear qualitative and quantitative research questions (or objectives)? | Yes, very clear. | Yes. | Yes. | No |
| Do the collected data allow for the research question (or objective) to be addressed? | Yes. | Yes | Yes. | No |
| Are the sources of qualitative data (archives, documents, informants, observations) relevant to address the research question (objective)? | Yes.* | Yes, selection clear, reason for not participating given.* | Yes, service users and healthcare professionals of mental health trusts.* | Not clear. |
| Is the process for analysing qualitative data relevant to address the research question (objective)? | Yes, method, form and analysis very clear and well explained. * | Partially, the form of the data was not clear. | Yes, several sources each very clear and well explained.* | Not clear. |
| Is appropriate consideration given  to how findings relate to the context  (e.g., the setting in which the data were collected)? | Yes, implications for the type of study and wider scope discussed in detail.* | Yes.* | Yes.* | Yes.* |
| Is appropriate consideration given to how findings relate to researchers’ influence (e.g. through their interactions with participants)? | Yes, this is referenced.* | Yes, limitation acknowledged: facilitator was also the principle investigator increasing the potential for bias.* | No, not addressed or discussed. | No, not addressed or discussed. |

**B: Application of MMAT criteria to quantitative randomized controlled (trials):** **first author (reference)** **and score (indicated by number of stars)**

| Study    Question | Schneiderhan (3)* |
| --- | --- |
| Are there clear qualitative and quantitative research questions (or objectives)? | Yes, very clear. |
| Do the collected data allow address the research question (objective)? | Yes |
| Is there a clear description of the randomization (or an appropriate sequence generation)? | Yes, block randomisation method used.* |
| Is there a clear description of the allocation concealment (or blinding when applicable)? | Partially, however, centralised call in system used to inform the investigator of the subject’s random group assignment. |
| Are there complete outcome data (80% or above)? | No, not for all parameters. |
| Is there low withdrawal/drop-out (below 20%)? | No, 22%. |

**C: Application of MMAT criteria (modified) to quantitative non-randomised studies (i) : first author (reference)** **and score (indicated by number of stars)**

| Study    Question | Runcie (23)*** | Barnes (24)**** | Schneiderhan (1)**** | Lizer (2)* |
| --- | --- | --- | --- | --- |
| Are there clear qualitative and quantitative research questions (or objectives)? | Yes | Yes, very clear. | Yes, clear. | Yes, clear. |
| Do the collected data allow for the research question (or objective) to be addressed? | Yes | Yes. | Yes. | Yes. |
| Are participants (or organisations) recruited in such a way that it minimises selection bias? | Yes, all adult inpatients with specified diagnosis on adult inpatient ward between specified dates.* | Yes, measures taken to reduce the potential for selection bias.* | Yes, all patients under care of a psychiatry clinic.* | No, methods of recruitment are not clear so don’t know if there was bias or not. Risk of bias not discussed. |
| Are measurements appropriate  (clear origin, or validity known, or standard instrument) regarding the exposure/intervention and outcomes? | Yes, protocol developed with expert guidance/advice. * | Yes, based on published consensus statements and evidence-based clinical guidelines.* | Yes, as per national guidline.* | Yes* |
| In the pre and post study groups are the participants comparable, or do researchers take into account (control for) the difference between or any contamination that may have occurred? | Partially - patients admitted more than once during study period. But no demographic data provided | Yes.* | Yes, acknowledged in limitations.* | Did not account or control for any contamination that might have occurred. |
| Are there complete outcome data (80% or above), and, when applicable, an acceptable response rate (60% or above), or an acceptable follow-up rate for cohort studies (depending on the duration of follow-up)? | Yes, notes available for 89% of eligible patients.* | Yes.* | Yes, as analysis of held administrative data.* | No, outcome data only reported for 74% of participants |

**C: Application of MMAT criteria (modified) to quantitative non-randomised studies (ii): first author (reference)** **and score (indicated by number of stars)**

| Study    Question | DelMonte (25)*** | McCleeary-Monthei (26)** | Kjeldsen (27)**** | Ramanuj (28)**** |
| --- | --- | --- | --- | --- |
| Are there clear qualitative and quantitative research questions (or objectives)/ | Yes, clear. | Yes, clear. | Yes, clear. | Yes, clear |
| Do the collected data allow for the research question (or objective) to be addressed? | Yes. | Yes. | Yes. | Yes. |
| Are participants (or organisations) recruited in such a way that it minimises selection bias? | Yes, all patients admitted to inpatient unit.* | Yes, all patients admitted to inpatient unit.* | Yes, all patients admitted to inpatient ward.* | Yes, all patients admitted to inpatient ward.* |
| Are measurements appropriate (clear origin, or validity known, or standard instrument) regarding the exposure/intervention and outcomes? | Partially, however, did not justify clearly enough why only two parameters from national guidance chosen. | Yes, based on national recognised guideline.* | Yes, based on clinical guidelines developed by expert group.* | Yes, based on local trust and published guidelines.* |
| In the pre and post study groups are the participants comparable, or do researchers take into account (control for) the difference between or any contamination that may have occurred? | Yes, discussed that [re and post groups comparable.* | No, not addressed or discussed. | Yes, issue of longer admission duration in one group discussed.* | Yes, issue of closure of one ward and catchment area discussed.* |
| Are there complete outcome data  (80% or above), and, when applicable, an acceptable response rate (60% or above), or an acceptable follow-up rate for cohort studies (depending on the duration of follow-up)? | Yes, as analysis of  held administrative data.* | No, not addressed or discussed. | Yes, as analysis of held administrative data.* | Yes, as analysis of held administrative data.* |

**C: Application of MMAT criteria (modified) to quantitative non-randomised studies (iii): first author (reference)** **and score (indicated by number of stars)**

| Study    Question | Sud (29) *** | Koffarnus (30)**** | Barnes (31)**** | Fischler (32)**** |
| --- | --- | --- | --- | --- |
| Are there clear qualitative and quantitative research questions (or objectives)? | Yes, clear. | Yes, clear. | Yes, very clear. | Yes, very clear. |
| Do the collected data allow for the research question (or objective) to be addressed? | Yes. | Yes | Yes. | Yes. |
| Are participants (or organisations) recruited in such a way that it minimises selection bias? | Yes, any patient under the care of inpatient, community or early intervention team/care in NHS Trust. | Yes, all patients screened in  a sequential manner for inclusion, with the first 60 patients meeting the criteria in each group included in the study..* | Yes, measures taken to reduce the potential for selection bias.* | Yes, all patients admitted to inpatient ward.* |
| Are measurements appropriate (clear origin, or validity known, or standard instrument) regarding the exposure/intervention and outcomes? | Yes, based on national quality improvement guideline.* | Yes, based on national recognised guideline.* | Yes, based on published consensus statements and evidence-based clinical guidelines.* | Yes, published expert guidelines reviewed with recognised framework.* |
| In the pre and post study groups are the participants comparable, or do researchers take into account (control for) the difference between or any contamination that may have occurred? | No, not addressed or discussed. | Yes, addressed and discussed.* | Yes.* | Yes, limitations and impact of other factors that may have contributed to the results discussed e.g artefact of improved documenatation.* |
| Are there complete outcome data  (80% or above), and, when applicable, an acceptable response rate (60% or above), or an acceptable follow-up rate for cohort studies (depending on the duration of follow-up)? | Yes, all patient data followed up and complete and reviewed by external body.* | Yes, as analysis of held administrative data.* | Yes.* | Yes, all data made available and accessible via electronic system.* |

**C: Application of MMAT criteria (modified) to quantitative non-randomised studies (iv): first author (reference)** **and score (indicated by number of stars)**

| Study    Question | Sasson (33)**** | Pena (39)** |
| --- | --- | --- |
| Are there clear qualitative and quantitative research questions (or objectives)? | Yes, clear. | Yes, clear |
| Do the collected data allow for the research question (or objective) to be addressed? | Yes. | Yes |
| Are participants (or organisations) recruited in such a way that it minimises selection bias? | Yes, any resident admitted to residential home.* | Yes* |
| Are measurements appropriate (clear origin, or validity known, or standard instrument) regarding the exposure/intervention and outcomes? | Yes, appropriate for primary and secondary outcomes.* | Yes* |
| In the pre and post study groups are the participants comparable, or do researchers take into account (control for) the difference between or any contamination that may have occurred? | Yes, no contamination possible as all care from one service.* | No, no discussion of contamination at all. Demographic data for pre and post not reported so could not also therefore account for differences between them as not known. |
| Are there complete outcome data  (80% or above), and, when applicable, an acceptable response rate (60% or above), or an acceptable follow-up rate for cohort studies (depending on the duration of follow-up)? | Yes, as analysis of data from care at one site.* | No, outcome data could only be reported for 60% of the patient participants. (Those who were referred and attended for at least one appointment). |

**C: Application of MMAT criteria to quantitative non-randomised studies (i): first author (reference)** **and score (indicated by number of stars)**

| Study    Question | Taveira (46) **** | Cohen (47)**** | Bozymski (40) ** |
| --- | --- | --- | --- |
| Are there clear qualitative and quantitative research questions (or objectives)? | Yes, very clear. | Yes, very clear. | Yes. |
| Do the collected data allow for the research question (or objective) to be addressed? | Yes. | Yes. | Yes. |
| Are participants (or organisations) recruited in such a way that it minimises selection bias? | Yes, referral made independent of mental health condition.* | Yes, discharged from cardiovascular risk reduction clinic.* | Yes, all patients at any of the two sites.* |
| Are measurements appropriate (clear origin, or validity known, or standard instrument; and absence of contamination between groups when appropriate) regarding the exposure/intervention and outcomes? | Yes, recognised risk tool used to calculate cardiovascular risk and patient status at point of referral and impact of this discussed in detail.* | Yes, based on national standard.* | Yes, in line with national guidelines.* |
| In the groups being compared (exposed vs. non-exposed; with intervention vs. without; cases vs. controls), are the participants comparable, or do researchers take into account (control for) the difference between these groups? | Yes, discussed in detail.* | Yes. | No, not fully there were significant differences between the groups including numbers compared, frequency of follow up during study period. |
| Are there complete outcome data  (80% or above), and, when applicable, an acceptable response rate (60% or above), or an acceptable follow-up rate for cohort studies (depending on the duration of follow-up)? | Yes, all followed up as analysis of held administrative data.* | Yes. | Not for both groups; 58% follow up in one group and 88% follow up in the other group. |

**D: Application of MMAT criteria to quantitative descriptive studies (i): first author (reference)** **and score (indicated by number of stars)**

| Study    Question | MacHaffie (42)* | Gable (43)** | Lucca (44)*** | Porras- Segovia (49)**** |
| --- | --- | --- | --- | --- |
| Are there clear qualitative and quantitative research questions (or objectives)? | Yes, clear | Yes. | Yes | Yes. |
| Do the collected data allow for the research question (or objective) to be addressed? | Yes | Yes. | Yes | Yes. |
| Is the sampling strategy relevant to address the quantitative research question (quantitative aspect of the mixed methods question)? | Partially, sampling bias due to the use of convenience sampling. | Yes, all patients under care of a team.* | Yes.* | Yes.* |
| Is the sample representative of the population understudy? | Partially, no reference to refusal or participation rates or follow-up engagement – therefore unknown. | Partially, no reference to refusal or participation rates or follow-up engagement – therefore unknown. | Partially, no reference to refusal or participation rates or follow-up engagement – therefore unknown. | Yes.* |
| Are measurements appropriate (clear origin, or validity known, or standard instrument)? | Yes.* | Yes.* | Yes.* | Yes, all relevant measurements taken in line with guidelines.* |
| Is there an acceptable response rate (60% or above)? | No, there is no reference to this. | No reference to this, cannot tell. | Yes, 97% of suspected adverse drug reactions evaluated. 90% of those who gained weight were enrolled onto weigh management programme. | Yes, full set of outcome data.* |

**D: Application of MMAT criteria to quantitative descriptive studies (ii): first author (reference)** **and score (indicated by number of stars)**

| Study    Question | Bozymski (48)*** | Sharma (16,17)***(two papers from same study) | Raynsford (45) |
| --- | --- | --- | --- |
| Are there clear qualitative and quantitative research questions (or objectives)? | Yes. | Yes, very clear. | Yes. |
| Do the collected data allow for the research question (or objective) to be addressed? | Yes | Yes. | Yes. |
| Is the sampling strategy relevant to address the quantitative research question (quantitative aspect of the mixed methods question)? | Yes.* | No, sampling bias due to the use of convenience sampling. | No, inclusion and exclusion criteria not clearly stated. Also reasons why certain GP practices chose to participate whilst others did not was not explained. |
| Is the sample representative of the population understudy? | Yes, all clients in early intervention care.* | Yes, appropriate as used in other studies and formulated by expert group.* | Yes. Patients with SMI on mood stabiliser or antipsychotic on GP records.* |
| Are measurements appropriate (clear origin, or validity known, or standard instrument)? | Partly, not clear what the measurements to be measured are based on. | Yes, based on a smoking cessation guideline published by Royal College of GPs.* | Yes.* |
| Is there an acceptable response rate (60% or above)? | Yes, as analysis of held administrative data.* | Yes, response rate 80.4%.* | No follow up regarding on whether recommendations/interventions were acted upon or their impact. |

**E: Application of MMAT criteria to mixed-methods studies: first author (reference)** **and score (indicated by number of stars)**

| Study    Question | Ohlsen (18)* | Watkins (50)* | Lee (20)** | Health foundation (21)** |
| --- | --- | --- | --- | --- |
| Is there a clear mixed methods question (or objective)? | Yes, clear. | Yes. | Yes, clear. | Yes, clear |
| Do the collected data allow for the research question (or objective) to be addressed? | Yes. | Yes. | Yes. | Yes |
| Is the mixed methods research design relevant to address the qualitative and quantitative research questions (or objectives), or the qualitative and quantitative aspects of the mixed methods question (or objective)? | Partially, however, qualitative methods aren’t explained or discussed. | Partially, however, reasons for reporting qualitative aspect isn’t explained or discussed. | Yes. | Yes, however, the reasons or justification for using a mixed methods design isn’t discussed |
| Is the integration of qualitative and quantitative data (or results) relevant to address the research question (objective)? | No integration of data | No integration of data | Partial reference to this but not clear. | No integration of data |
| Is appropriate consideration given to the limitations associated with this integration, e.g., the divergence of qualitative and quantitative data (or results*) in a triangulation design? | Not relevant as there is no integration of the data. | Not relevant as there is no integration of the data. | No | Not relevant as there is no integration of the data. |
| Are the criteria for qualitative studies met? | a. No  b. No  c. Yes, reference to changing referral process, co-working, boundaries and clarity of roles within the team.*  d. No, no reference to researcher influence. | a. No  b. No.  c. Yes, but very briefly.*  d. No, no reference to researcher influence. | a. Yes.*  b. Partially, however, no data analysis undertaken.  c. Yes, context discussed.*  d. No, no reference to researcher influence. | a. Yes.*  b. No  c. Yes, reference to how the community pharmacists feedback is important in terms of their opinion about patient care.*  d. No, no reference to researcher influence. |
| Are the criteria for quantitative studies met? | a. Yes, referral from different settings.*  b. Yes, very very clear.*  c. A single group was used and therefore no contamination.*  d. No reference to refusal or participation rates. There are complete outcome data. | a. Yes.*  b. Yes.*  c. Yes, discussed as a limitation.*  d. Yes, 26% drop out accounted for in detail and complete follow up data on remaining as analysis of held administrative data.* | a. Yes.*  b. Partially, however, did not justify clearly enough why only two parameters from national guidance chosen.  c. Yes.*  d. Yes.* | a. Yes*  b. Yes*  c. No, not discussed at all.  d. No, only 70% |

**References**

1. Schneiderhan ME, Batscha CL, Rosen C. Assessment of a point-of-care metabolic risk screening program in outpatients receiving antipsychotic agents. Pharmacotherapy. 2009 Aug;29(8):975–87.

2. Lizer MH, Parnapy Jawaid SA, Marsh W, Mogili L. The impact of a pharmacist assisted clinic upon medication adherence and quality of life in mental health patients. Pharm Pract (Granada) [Internet]. 2011;9(3):122–7. Available from: http://gateway.proquest.com/openurl?ctx_ver=Z39.88-2004&res_id=xri:pqm&req_dat=xri:pqil:pq_clntid=47823&rft_val_fmt=ori/fmt:kev:mtx:journal&genre=article&issn=1885-642X&volume=9&issue=3&spage=122

3. Schneiderhan ME, Shuster SM, Davey CS. Twelve-month prospective randomized study of pharmacists utilizing point-of-care testing for metabolic syndrome and related conditions in subjects prescribed antipsychotics. Prim Care Companion J Clin Psychiatry. 2014;16(5).

4. NHS Centre for Reviews and Dissemination Systematic Reviews. CRD’s Guidance for Undertaking Reviews in Healthcare. York: CRD University of York; 2009.

5. Higgins JP, Green S. Cochrane Handbook for Systematic Reviews of Interventions Version 5.2 [Internet]. The Cochrane Collaboration. 2017. Available from: http://training.cochrane.org/handbook

6. Methley AM, Campbell S, Chew-Graham C, McNally R, Cheraghi-Sohi S. PICO, PICOS and SPIDER: A comparison study of specificity and sensitivity in three search tools for qualitative systematic reviews. BMC Health Serv Res. 2014;14(1):579.

7. Noyes J. Never mind the qualitative feel the depth! The evolving role of qualitative research in Cochrane intervention reviews. J Res Nurs. 2010;16(6):525–34.

8. Booth A. EVIDENT Guidance for Reviewing the Evidence: A Compendium of Methodological Literature and Websites [Internet]. 2016 [cited 2018 Jul 1]. Available from: www.academia.edu/21598179/EVIDENT_Guidance_for_Reviewing_the_Evidence_a_compendium_of_methodological_literature_and_websites

9. Jootun D. Reflexivity- Promoting rigor in qualitative research. Nurs Stand. 2009;23(23):42–6.

10. O’Cathain A, Thomas K. Combining qualitative and quantitative methods. In: Pope C MN, editor. Qualitative Research in Health Care. Third. Oxford: Blackwell Publishing; 2006. p. 102–11.

11. Sheldon TA. Making evidence synthesis more useful for management and policy-making. J Health Serv Res Policy [Internet]. 2005;10(1_suppl):1–5. Available from: http://journals.sagepub.com/doi/10.1258/1355819054308521

12. Pluye P, Gagnon MP, Griffiths F, Johnson-Lafleur J. A scoring system for appraising mixed methods research, and concomitantly appraising qualitative, quantitative and mixed methods primary studies in Mixed Studies Reviews. Int J Nurs Stud. 2009;46(4):529–46.

13. Pluye P, Robert E, Cargo M, Bartlett G. Proposal: A mixed methods appraisal tool for systematic mixed studies reviews [Internet]. Montréal: McGill University. 2011 [cited 2018 Feb 25]. Available from: http://mixedmethodsappraisaltoolpublic.pbworks.com

14. Pace R, Pluye P, Bartlett G, Macaulay A, Salsberg J, Jagosh J, et al. Reliability of a tool for concomitantly appraising the methodological quality of qualitative, quantitative and mixed methods research: a pilot study. 38th Annual Meeting of the North American Primary Care Research Group (NAPCRG. Seattle, USA; 2010.

15. Pace R, Pluye P, Bartlett G, Macaulay AC, Salsberg J, Jagosh J, et al. Testing the reliability and efficiency of the pilot Mixed Methods Appraisal Tool (MMAT) for systematic mixed studies review. Int J Nurs Stud. 2012;49(1):47–53.

16. Sharma R, Meurk C, Bell S, Ford P, Gartner C. Australian health practitioners’ adherence to the 5A’s of smoking cessation and barriers to delivering smoking cessation assistance to smokers with severe mental illness. In: Conference: Society for Research on Nicotine and Tobacco. Florence, Italy: The University Of Queenslands; 2017.

17. Sharma R, Meurk C, Bell S, Ford P, Gartner C. Australian mental health care practitioners’ practices and attitudes for encouraging smoking cessation and tobacco harm reduction in smokers with severe mental illness. Int J Ment Health Nurs. 2018;27(1):247–57.

18. Ohlsen RI, Peacock G, Smith S, Ward M, Jackson A. Developing a service to monitor and improve physical health in people with serious mental illness. J Psychiatr Ment Health Nurs. 2005;12(5):614–9.

19. Watkins Bruce R Winchester Nancy C Brahm Nicole B Washington SC, Watkins SC, Candidate P, Winchester BR, Brahm NC, Professor C, et al. Development of a Medication Monitoring System for an Integrated Multidisciplinary Program of Assertive Community Treatment (IMPACT) Team Recommended Citation Development of a Medication Monitoring System for an Integrated Multidisciplinary Program of Asse. Inov Pharm. 2012;3(4):Article 99.

20. Lee J, Dalack GW, Casher MI, Eappen SA, Bostwick JR. Persistence of metabolic monitoring for psychiatry inpatients treated with second-generation antipsychotics utilizing a computer-based intervention. J Clin Pharm Ther. 2016 Apr;41(2):209–13.

21. The Health Foundation. Improving Physical health care for patients with psychosis (PHCP) through collaborative working with local community pharmacies. North East London NHS Foundatioin Trust [Internet]. 2018. Available from: https://www.health.org.uk/programmes/innovating-improvement/projects/improving-physical-health-care-patients-psychosis-phcp

22. Creswell J, Clark V. Designing and conducting mixed-methods research. Thousand Oaks, CA: Sage. The Sage handbook of qualitative research. 2007.

23. Runcie O, Boilson M HR. Monitoring weight and blood glucose in in-patients: how helpful is a protocol? Psychiatrist. 2007;31:88–91.

24. Barnes TRE, Paton C, Hancock E, Cavanagh M-RR, Taylor D, Lelliott P. Screening for the metabolic syndrome in community psychiatric patients prescribed antipsychotics: A quality improvement programme. Acta Psychiatr Scand. 2008;118(1):26–33.

25. DelMonte MT, Bostwick JR, Bess JD, Dalack GW. Evaluation of a computer-based intervention to enhance metabolic monitoring in psychiatry inpatients treated with second-generation antipsychotics. J Clin Pharm Ther. 2012;37(6):668–73.

26. McCleeary-Monthei E, Kutscher EC. Evaluation of lipid and glucose monitoring after implementation of a pharmacist initiated antipsychotic monitoring form. J Pharm Pract. 2012;25(2):281.

27. Kjeldsen LJ, Hansen PS, Kristensen AMF, Christensen A, Sørensen CH, Nielsen B. Outreach visits by clinical pharmacists improve screening for the metabolic syndrome among mentally ill patients. Nord J Psychiatry. 2013;67(4):249–57.

28. Ramanuj PP. Improving blood and ECG monitoring among patients prescribed regular antipsychotic medications. Ment Health Fam Med. 2013;10(1):29–36.

29. Sud D. Improving physical healthcare to reduce premature mortality in people with serious mental illness (SMI): the Leicestershire Physical Health Register (LPHR). In: LPT NHS Trust Annual General Meeting. 2017.

30. Koffarnus RL, Mican LM, Lopez DA, Barner JC. Evaluation of an inpatient psychiatric hospital physician education program and adherence to American Diabetes Association practice recommendations. Am J Health Syst Pharm. 2015;73(Suppl 2):S57-62.

31. Barnes TRE, Bhatti SF, Adroer R, Paton C. Screening for the metabolic side effects of antipsychotic medication: Findings of a 6-year quality improvement programme in the UK. BMJ Open. 2015;5(10):e007633.

32. Fischler I, Riahi S, Stuckey MI, Klassen PE. Implementation of a clinical practice guideline for schizophrenia in a specialist mental health center: An observational study. BMC Health Serv Res [Internet]. 2016;16(1):1–11. Available from: http://dx.doi.org/10.1186/s12913-016-1618-9

33. Sasson E, James M, Wubshet B, Todorov D, Cohen H. Implementing Psychopharmacology Rounds in a Nursing Facility to Improve Antipsychotic Usage. Consult Pharm. 2017;32(6):352–9.

34. Peña A, DeJongh B, Haas M HM. Overcoming barriers to monitoring patients taking second-generation antipsychotics. Ment Heal Clin. 2018;8(2):49–55.

35. Quirk A, Chee S, Patterson S, Snowdon C, Lemmey S, Tooke B, et al. An Evaluation of the Implementation of the Lester Tool 2014 in Psychiatric Inpatient Settings [Internet]. London; 2016. Available from: https://www.rcpsych.ac.uk/pdf/eLester final report 16.03.16.pdf

36. Taylor DA, Sutton J, Dawson HE. Evaluating the Pharmacist Provision of Clozapine Services. Bath: University of Bath; 2011.

37. Shanker S. Managing severe mental illness in primary care. CPAs in GP surgeries. Personal communication. Powerpoint presentation. Walsall. 2016.

38. McMorris T, Sweet G, Sullivan CJ, Washington NB, Brahm N. A design and focus group evaluation of dietary choices tools for an underserved population. Ment Heal Clin. 2016;6(2):101–8.

39. Hor ESL, Subramaniam S, Koay JM, Bharathy A, Vasudevan U, Panickulam JJ, et al. Improving metabolic monitoring in patients maintained on antipsychotics in Penang, Malaysia. Australas Psychiatry. 2016;

40. Bozymski K, Banker K, Lum C, Ruekert L, Cunningham E, Khan S, et al. Justifying the implementation of a collaborative drug therapy management (CDTM) protocol in an outpatient psychiatric clinic: A retrospective chart review. Vol. 28, Journal of Pharmacy Practice. [(Banker, Lum, Ruekert, Cunningham, Khan, Covington) Community Health Network, United States]: SAGE Publications Inc.; 2015.

41. Eccles M, Grimshaw J, Campbell M, Ramsay C. Research designs for studies evaluating the effectiveness of change and improvement strategies. Qual Saf Heal Care [Internet]. 2003;12(1):47–52. Available from: http://qualitysafety.bmj.com/lookup/doi/10.1136/qhc.12.1.47

42. MacHaffie S. Health promotion information: Sources and significance for those with serious and persistent mental illness. 2002 Dec;16(6):263–74. Available from: https://www.scopus.com/inward/record.uri?eid=2-s2.0-0036981382&doi=10.1053%2Fapnu.2002.37281&partnerID=40&md5=2287658668e47a77e54454fa11402995

43. Gable KN, Stunson MJ. Clinical pharmacist interventions on an assertive community treatment team. Community Ment Heal J [Internet]. 2009/10/07. 2010;46(4):351–5. Available from: https://www.ncbi.nlm.nih.gov/pubmed/19809876

44. Lucca JM, Madhan R, Parthasarathi G, Ram D. Identification and management of adverse effects of antipsychotics in a tertiary care teaching hospital. J Res Pharm Pract. 2014;3(2):46–50.

45. Raynsford J, Dada C, Stansfield D, Cullen T. Impact of a specialist mental health pharmacy team on medicines optimisation in primary care for patients on a severe mental illness register: a pilot study. Eur J Hosp Pharm. 2018;

46. Taveira TH, Pirraglia PA, Cohen LB, Wu W-CW-CCW-CC, W.-C. W, Wu W-CW-CCW-CC. Efficacy of a pharmacist-led cardiovascular risk reduction clinic for diabetic patients with and without mental health conditions. Prev Cardiol [Internet]. 2008;11(4):195–200. Available from: http://onlinelibrary.wiley.com/doi/10.1111/j.1751-7141.2008.00008.x/full

47. Cohen LB, Taveira TH, Wu WC, Pirraglia PA. Maintenance of risk factor control in diabetic patients with and without mental health conditions after discharge from a cardiovascular risk reduction clinic. Ann Pharmacother. 2010;44(7–8):1164–70.

48. Bozymski KM, Whitten JA, Blair ME, Overley AM, Ott CA. Monitoring and Treating Metabolic Abnormalities in Patients with Early Psychosis Initiated on Antipsychotic Medications. Community Ment Health J. 2017;54(Suppl 1):1–8.

49. Porras-Segovia A, Krivoy A, Horowitz M, Thomas G, Bolstridge M, Ion D, et al. Rapid-onset clozapine-induced loss of glycaemic control: case report. BJPsych open. 2017;3(3):138–40.

50. Watkins SC, Winchester BR, Brahm N, Washington NB. Development of an antipsychotic monitoring program for a Program of Assertive Community Treatment (PACT) team. J Pharm Pract. 2012;25 (2):264.
