# Supplementary material for: The role of pharmacy in the management of cardiometabolic risk, metabolic syndrome and related diseases in severe mental illness: a mixed-methods systematic literature review
Source: Syst Rev. 2021 Mar 31;10:92. doi: 10.1186/s13643-021-01586-9 (PMC8015120; doi:10.1186/s13643-021-01586-9)
Supplement: Supplementary file 3 — Additional file 3. Effective Practice and Organisation of Care (EPOC) Taxonomy of interventions. [file 13643_2021_1586_MOESM3_ESM.docx]

**Additional file 3:**

**Effective Practice and Organisation of Care (EPOC) Taxonomy of interventions.**

Based on the original tool published in 2002 (1)

Cochrane Effective Practice and Organisation of Care Review Group. Cochrane Effective Practice and Organisation of Care Review Group - Data collection checklist; 2002. [consulted 01/05/2018]. [**http://methods.cochrane.org/sites/methods.cochrane.org.bias/files/public/uploads/EPOC%20Data%20Collection%20Checklist.pdf**](http://methods.cochrane.org/sites/methods.cochrane.org.bias/files/public/uploads/EPOC%20Data%20Collection%20Checklist.pdf)

This review included interventions that fell under the following types as per the EPOC taxonomy (professional, financial, organizational, patient centred, regulatory).

**2.1 EPOC Taxonomy of interventions**

| ***Professional interventions*** |
| --- |
| - **Distribution of educational materials (**Distribution of published or printed recommendations for clinical care, including clinical practice guidelines, audio-visual materials and electronic publications. The materials may have been delivered personally or through mass mailings). - **Educational meetings** (Health care providers who have participated in conferences, lectures, workshops or traineeships). - **Local consensus processes** (Inclusion of participating providers in discussion to ensure that they agreed that the chosen clinical problem was important and the approach to managing the problem was appropriate). - **Educational outreach visits** (Use of a trained person who met with providers in their practice settings to give information with the intent of changing the provider’s practice. The information given may have included feedback on the performance of the provider(s). - **Local opinion leaders** (Use of providers nominated by their colleagues as ‘educationally influential’. The investigators must have explicitly stated that their colleagues identified the opinion leaders). - **Patient mediated interventions** (New clinical information (not previously available) collected directly from patients and given to the provider e.g. depression scores from an instrument). - **Audit and feedback** (Any summary of clinical performance of health care over a specified period of time. The summary may also have included recommendations for clinical action. The information may have been obtained from medical records, computerized databases, or observations from patients). - **Reminders** (Patient or encounter specific information, provided verbally, on paper or on a computer screen, which is designed or intended to prompt a health professional to recall information. This would usually be encountered through their general education; in the medical records or through interactions with peers, and so remind them to perform or avoid some action to aid individual patient care. Computer aided decision support and drugs dosage are included). - **Marketing** (Use of personal interviewing, group discussion (‘focus groups’), or a survey of targeted providers to identify barriers to change and subsequent design of an intervention that addresses identified barriers). - **Mass media** (1- varied use of communication that reached great numbers of people including television, radio, newspapers, posters, leaflets, and booklets, alone or in conjunction with other interventions; 2- targeted at the population level). |
| ***Financial interventions*** |
| 1. **Provider:**  - **Fee-for-service** (provider has been paid for number and type of service delivered). - **Prepaid** (no other description). - **Capitation** (provider was paid a set amount per patient for providing specific care). - **Provider salaried service** (provider received basic salary for providing specific care). - **Prospective payment** (provider was paid a fixed amount for health care in advance). - **Provider incentives** (provider received direct or indirect financial reward or benefit for doing specific action). - **Institution incentives** (institution or group of providers received direct or indirect financial rewards or benefits for doing specific action). - **Provider grant/allowance** (provider received direct or indirect financial reward or benefit not tied to specific action). - **Institution grant/allowance** (institution or group of providers received direct or indirect financial reward or benefit not tied to specific action). - **Provider penalty** (provider received direct or indirect financial penalty for inappropriate behaviour). - **Institution penalty** (institution or group of providers received direct or indirect financial penalty for inappropriate behaviour). - **Formulary** (added or removed from reimbursable available products).  1. **Patient:**  - **Premium** (Patient payment for health insurance. It is important to determine if the patient paid the entire premium, or if the patient’s employer paid some of it. This includes different types of insurance plans). - **Co-payment** (Patient payment at the time of health care delivery in addition to health insurance e.g. in many insurance plans that cover prescription medications the patient may pay 5 dollars per prescription, with the rest covered by insurance). - **User-fee** (Patient payment at the time of health care delivery). - **Patient incentives** (Patient received direct or indirect financial reward or benefit for doing or encouraging them to do specific action). - **Patient grant/allowance** (Patient received direct or indirect financial reward or benefit not tied to specific action). - **Patient penalty** (Patient received direct or indirect financial penalty for specified behaviour e.g. reimbursement limits on prescriptions). |
| ***Organizational interventions*** relates to the arrangement of healthcare provision. Interventions include provision of new service or change to healthcare professional roles e.g. enhancement of core day to day activities or providing care that may have otherwise been provided by other healthcare providers. This strategy would also include a pharmacist leading or becoming part of a multidisciplinary team where they were not before. They may liaise with other healthcare professionals to provide continuity of care between healthcare settings or to other HCPs. The setting up of a pharmacist clinic or service that patient can attend would also be include here. |
| 1. **Provider orientated interventions**  - **Revision of professional roles** (Also known as ‘professional substitution’, ‘boundary encroachment’ and includes the shifting of roles among health professionals. For example, nurse midwives providing obstetrical care; pharmacists providing drug counselling that was formerly provided by nurses and physicians; nutritionists providing nursing care; physical therapists providing nursing care. (Also includes expansion of role to include new tasks). - **Clinical multidisciplinary teams** (creation of a new team of health professionals of different disciplines or additions of new members to the team who work together to care for patients). - **Formal integration of services** (bringing together of services across sectors or teams or the organization of services to bring all services together at one time also sometimes called ‘seamless care’). - **Skill mix changes** (changes in numbers, types or qualifications of staff). - **Continuity of care** (including one or many episodes of care for inpatients or outpatients): - Arrangements for follow-up; - Case management (including co-ordination of assessment, treatment and arrangement for referrals). - **Satisfaction of providers** with the conditions of work and the material and psychic rewards (e.g. interventions to ‘boost morale’). - **Communication and case discussion** between distant health professionals (e.g. telephone links; telemedicine; there is a television/video link between specialist and remote nurse practitioners).  1. **Patient orientated interventions**  - **Mail order pharmacies** (e.g. compared to traditional pharmacies). - Presence and functioning of adequate **mechanisms for** dealing with patients’ **suggestions and complaints.** - **Consumer participation** in governance of health care organization. - Other (other categories to be agreed in consultation with the EPOC editorial team).  1. **Structural interventions**  - **Changes to the setting/site of service delivery** (e.g. moving a family planning service from a hospital to a school). - **Changes in physical structure, facilities and equipment** (e.g. change of location of nursing stations, inclusion of equipment where technology in question is used in a wide range of problems and is not disease specific, for example an MRI scanner). - **Changes in medical records systems** (e.g. changing from paper to computerized records, patient tracking systems). - **Changes in scope and nature of benefits and services.** - **Presence and organization of quality monitoring mechanisms.** - **Ownership, accreditation, and affiliation status of hospitals and other facilities.** - **Staff organization.** |
| ***Patient-centred interventions*** this would include any intervention directed primarily at individuals such as direct provision of advice or information e.g. smoking cessation advice or strategies for weight management. (Screening for healthcare parameters e.g. taking blood test or checking BP is not included in this category). |
| - **Education/educational materials** - **Peer support** - **Support for self-management**. Including telephone and telemedicine interventions with predominant patients elements (with focus on self-management) - **Behaviour change** - **Reminders** e.g. reminder card - **Motivational counselling** |
| ***Regulatory interventions*** Any intervention that aims to change health services delivery or costs by regulation or law. (These interventions may overlap with organisational and financial interventions.) |
| - **Changes in medical liability** - **Management of patient complaints** - **Peer review** - **Licensure** |

1. Cochrane Effective Practice and Organisation of Care (EPOC) Review Group. Data collection checklist. Cochrane Effective Practice and Organisation of Care. 2002.
